# The DNA-PK inhibitor AZD7648 alone or combined with pegylated liposomal doxorubicin in patients with advanced cancer: results of a first-in-human Phase I/IIa study

**DOI:** 10.1038/s41416-025-03053-x

**Published:** 2025-05-17

**Authors:** Timothy A. Yap, Patricia LoRusso, Rowan E. Miller, Rebecca Kristeleit, Amanda G. Paulovich, Stephen McMorn, Lenka Oplustil O’Connor, Benedetta Lombardi, Paola Marco-Casanova, Eric T. Gangl, Bharat Patel, Mark J. O’Connor, Emma Dean, Roman Zviezdin, Ruth Plummer

**Affiliations:** 1 University of Texas MD Anderson Cancer Center, Houston, TX USA; 2 Yale Cancer Centre, Yale University, New Haven, CT USA; 3 University College London Hospitals NHS Foundation Trust, London, UK; 4 Department of Oncology, Guy’s and St Thomas’ NHS Foundation Trust and King’s College London, London, UK; 5 Fred Hutchinson Cancer Center, Seattle, WA USA; 6 Oncology R&D, AstraZeneca, Cambridge, UK; 7 Clinical Pharmacology and Safety Sciences, Biopharmaceuticals R&D, AstraZeneca, Waltham, MA USA; 8 Newcastle University and Northern Centre for Cancer Care, Newcastle Hospitals NHS Trust, Newcastle Upon Tyne, UK

**Keywords:** Cancer, Cancer

## Abstract

**Background:**

Upregulation of DNA-dependent protein kinase (DNA-PK) is associated with poor prognosis and decreased response to DNA-damaging agents across cancer types. A Phase I/IIa study (NCT03907969) investigated the highly potent, selective DNA-PK inhibitor AZD7648 as monotherapy or combined with pegylated liposomal doxorubicin (PLD) in patients with advanced cancer.

**Methods:**

Thirty patients received escalating doses of AZD7648 as monotherapy (*n* = 14), starting at 5 mg QD, or with PLD 40 mg/m^2^ (*n* = 16). The primary objective was safety and tolerability.

**Results:**

AZD7648 monotherapy was administered at 5–160 mg BID. The most frequent class of adverse events was gastrointestinal disorders (9/14 patients, 64.3%); one patient (160 mg BID) experienced dose-limiting toxicities (DLTs). No responses to AZD7648 monotherapy were observed. The maximum dose of combination therapy was AZD7648 40 mg QD days 1–7 + PLD every 28 days. 13/16 patients (81.3%) experienced gastrointestinal disorders and 11/16 (68.8%) patients had anaemia. Three patients experienced DLTs (two at AZD7648 20 mg QD 7 days + PLD; one at AZD7648 30 mg QD 7 days + PLD). Limited efficacy was observed, with one RECIST partial response.

**Discussion:**

Toxicity of AZD7648 + PLD was greater than expected and antitumour activity was limited, leading to early study termination.

## Background

Cellular DNA damage response (DDR) pathways maintain genomic stability and promote cellular survival. However, in cancer cells, these pathways are often defective [1]. This leads to genomic instability, neoplastic transformation and proliferation, resulting in the accumulation of DNA lesions that induce DNA replication stress and the formation of double-stranded breaks (DSBs) [1]. DSBs are the most cytotoxic type of DNA damage; they can trigger both cell cycle arrest and cell death [1].

Different forms of DNA damage trigger different repair mechanisms [1]. DSBs are repaired by both non-homologous end joining (NHEJ) and homologous recombination during interphase (G1, S and G2), while microhomology-mediated end joining is the sole DSB repair mechanism in mitosis [1]. DNA-dependent protein kinase (DNA-PK) is a member of the phosphatidylinositol-3-kinase-related kinase family and is a key modulator of NHEJ [2]. DNA-PK is upregulated in various tumour types; this upregulation is associated with a poorer clinical prognosis, decreased response to DNA-damaging agents and therapeutic resistance in various types of cancer [3–10]. Therefore, using a DNA-PK inhibitor to prevent cancer cells repairing DSBs could be an effective therapeutic strategy [4]. Synthetic lethal interactions have been identified between DNA-PK and multiple damage response factors, including ataxia-telangiectasia mutated (ATM) and homologous recombination proteins [11]. This suggests that DNA-PK inhibitors may have anticancer activity as monotherapy in ATM-deficient tumours, which is supported by preclinical data [11]. Furthermore, combining a DNA-PK inhibitor with a therapeutic modality that increases the incidence of DSBs, such as topoisomerase-II inhibition or radiation, is also a rational approach [12–14]. These observations support the clinical development of DNA-PK inhibitors [4, 13, 15].

AZD7648 is a novel, highly potent, selective DNA-PK inhibitor that has been shown to have activity as monotherapy in preclinical models [4, 11, 14]. Preclinical data also indicate that it has synergistic effects with the pegylated liposomal doxorubicin (PLD) [12], radiotherapy [13], and the PARP inhibitor olaparib [4, 16], potentially due to prevention of the repair of the DSBs caused by these therapies, resulting in cell death [3, 12]. PLD was selected as a combination partner based on available preclinical data and because it has similar efficacy to the uncapsulated form, but with reduced cardiotoxicity and haematotoxicity [17].

We report data from an open-label, multicentre Phase I/IIa study (NCT03907969) that investigated the safety, tolerability, pharmacokinetics, pharmacodynamics and preliminary efficacy of oral AZD7648 as monotherapy or in combination with PLD in patients with advanced cancer.

## Patients and methods

### Study design and patients

**Fig. 1:**
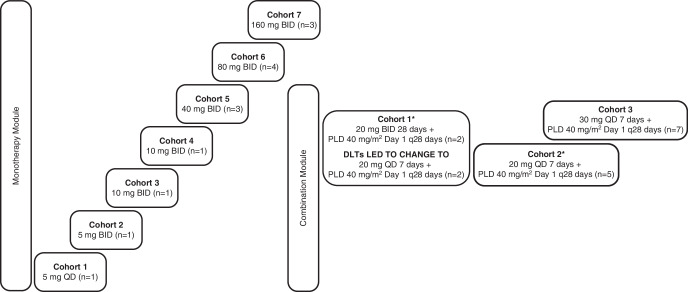
Study design. BID twice daily, PLD pegylated liposomal doxorubicin, QD daily. *The first two patients enrolled in Combination Module Cohort 1 and treated with AZD7648 20 mg QD for 28 days + PLD 40 mg/m^2^ on Day 1 of a 28-day cycle experienced DLTs. The SRC recommended a dose reduction to AZD7648 20 mg QD for 7 days and PLD 40 mg/m^2^ on Day 1 of a 28-day cycle for two patients. All patients in Cohort 2 received this same reduced dose of AZD7648 20 mg QD for 7 days and PLD 40 mg/m^2^ on Day 1 of a 28-day cycle as a separate cohort to confirm the safety before escalating to higher doses.

This was a modular, Phase I/IIa, open-label, multicentre study investigating the safety of the DNA-PK inhibitor AZD7648 as a monotherapy or in combination with PLD in patients with advanced malignancies. The modular design allowed dose escalation of AZD7648 as monotherapy (Monotherapy Module) and in combination with PLD (Combination Module) with intensive safety monitoring and oversight by a safety review committee (SRC) throughout (Fig. 1).

Patients included in this study had to be ≥18 years of age, with histological or cytological evidence of advanced malignancy, and progressive cancer at the time of study entry. They had an Eastern Cooperative Oncology Group Performance Status (ECOG-PS) of 0 or 1, and a life expectancy >12 weeks. Exclusion criteria included inadequate haematological function (haemoglobin <90 g/L with no blood transfusions or erythropoietin within 14 days; absolute neutrophil count <1500 cells/mm^3^ (<1.5 × 10^9^/L) with no haematopoietic growth factors within 14 days; platelet count <100,000/mm^3^ (<100 × 10^9^/L) with no platelet transfusions within 14 days of obtaining these values or before starting treatment); any unresolved toxicities from prior therapy; severe medical conditions including, but not limited to, cardiac dysfunction, uncontrolled diabetes mellitus, active infections, severe chronic obstructive pulmonary disease, or active malignancies; or cytotoxic treatment, non-cytotoxic treatment, biological treatment, or radiation therapy within a defined period before the first dose of study treatment (see Supplementary Materials pages 1–6 for full eligibility criteria).

### Ethics approval and consent to participate

The study was conducted in accordance with consensus ethical principles derived from international guidelines including the Declaration of Helsinki and Council for International Organizations of Medical Sciences International Ethical Guidelines, applicable International Conference on Harmonisation Good Clinical Practice Guidelines, and applicable laws and regulations. The protocol and any amendments and patient informed consent documents were submitted to and approved by institutional review boards/independent ethics committees. Written informed consent was obtained from all participants.

### Treatment

In the Monotherapy Module, the starting dose of AZD7648 was 5 mg once daily (QD) based on International Conference on Harmonisation S9 guidelines recommending a starting dose derived from the severely toxic dose in animals [18]. A single dose was administered during Cycle 0 and was followed by a drug washout period lasting 3–7 days. From Day 1 of Cycle 1, AZD7648 was dosed QD or twice daily (BID) on a 28-day cycle, with no interval between cycles. Dose escalation based on single-patient cohorts was allowed up to and including AZD7648 20 mg BID if the SRC recommended escalation and no grade ≥2 drug-related toxicity was observed. If either of these conditions was not met, the cohort would be expanded to a minimum of 3, and up to 6, patients in all subsequent cohorts. A Bayesian adaptive design, using all of the available data and considering the pharmacokinetics (PK) from a minimum of 2 or 3 patients enrolled into each dose cohort, was used from this point. Dose escalation was scheduled to be stopped at the maximum tolerated dose (MTD) (defined as 25% of patients in a cohort experiencing a dose-limiting toxicity [DLT] with the required precision [ratio of upper and lower 95% credible limits <5.0]), the maximum feasible dose (MFD) or the recommended Phase II dose (RP2D). Intra-patient dose escalation was permitted as long as predefined criteria were met, including that the planned next higher dose had to be declared tolerated by the SRC based on assessment of 2–6 evaluable patients.

Concomitant medications, including anti-emetic therapy, deemed necessary to provide adequate prophylactic or supportive care (except for those medications prohibited in the protocol, primarily anticancer therapy, herbal medications/supplements, medications known to interfere with CYP3A4 activity and live vaccines) could be prescribed by the investigator. However, the use of granulocyte colony-stimulating factor was not permitted for primary or secondary prophylaxis during Cycle 0 or Cycle 1 but could be prescribed as secondary prophylaxis from Cycle 2 onwards. Approximately 46 evaluable patients across 10 cohorts (*n* = 1–6 per cohort) were originally planned to provide an estimate of MTD in the Monotherapy Module. Determining the RP2D required data from a minimum of six evaluable patients.

In the Combination Module, a single dose of AZD7648 was administered during Cycle 0 and was followed by a drug washout period lasting 3–7 days. AZD7648 was administered daily for the first 7 days of each of the first six 28-day cycles. The starting dose of PLD was 40 mg/m^2^ intravenously once every 28 days for a maximum of 6 cycles. PLD dose could be escalated to 50 mg/m^2^ or de-escalated to 30 mg/m^2^ every 28 days with agreement from the SRC. AZD7648 could be continued after completion of six cycles of PLD, administered QD without interruption. Dose escalation was scheduled to be stopped at the MTD (defined as 30% of patients in a cohort experiencing a DLT with the required precision [ratio of upper and lower 95% credible limits <5.0]). Approximately 30 evaluable patients across five cohorts (*n* = 3–6 per cohort) were originally planned to provide an estimate of MTD.

### Objectives

The primary objective of this study was the safety and tolerability of AZD7648 alone and in combination with PLD. Secondary objectives included characterisation of the PK of AZD7648 following a single dose and at steady state after multiple doses, both as monotherapy and in combination with PLD, and preliminary assessment of antitumour activity. Characterisation of the pharmacodynamics (PD) of AZD7648 as monotherapy and in combination with PLD, following single and multiple doses, was an exploratory objective.

### Assessments

Treatment-emergent adverse events (TEAEs) were monitored continuously and summarised using MedDRA system organ class (SOC), MedDRA preferred term (PT) and CTCAE grade. Dose-limiting toxicities were predefined TEAEs (see Supplementary Materials page 7–8) occurring from the first dose of AZD7648 until the end of Cycle 1 (inclusive of any non-dosing days), the period when the major potential toxicities were anticipated to occur.

The preliminary antitumour activity of AZD7648 alone and in combination with PLD was assessed based on radiological response using computed tomography or magnetic resonance imaging performed every 8 weeks and evaluated using response evaluation criteria in solid tumours (RECIST) v1.1. The imaging method used at baseline had to be used at all follow-up visits. The following parameters were estimated: percentage best change in target lesion; duration of response; objective response rate (ORR); and progression-free survival (PFS).

Single-dose PK parameters were determined from venous blood samples (2 mL) for AZD7648 and its metabolites and for PLD (see Supplementary Materials Tables 1 and 2 for the sampling schedule). Urine PK samples were collected at Cycle 0, Day 1 and at Cycle 1, Day 8 at the following timepoints: pre-dose (Cycle 0, Day 1 only), 0 to 8 h post dose and 8 to 24 h post-dose. PK analysis of plasma and urine concentration data for AZD7648 and its metabolites and PLD (plasma only) was conducted by Covance Bioanalytical Laboratory Services Inc, (Madison, WI, USA) using a validated bioanalytical method, and PK parameters were calculated using Pheonix^®^ WinNonlin^®^ version 8.1 or higher. Maximum observed concentration (C_max_) and time to reach C_max_ (t_max_) were determined directly by inspection of the concentration-time profiles. Terminal half-life slope (t_½λz_) was calculated as ln2/terminal elimination rate constant (λz). Area under the plasma concentration time curve from zero to last measurable concentration (AUC_last_) was calculated using the linear up/log down trapezoidal rule. AUC_last_ was extrapolated to infinity using λz to obtain overall exposure (AUC_inf_). The amount of drug excreted unchanged in the urine (e.g. Ae0-t, Aeτ) for any given dosing interval was determined by multiplying the concentration of drug determined in the urine sample by the volume of the sample collected in that period.

To characterise the PD of AZD7648 following a single dose and at steady state after multiple doses, biomarkers of the DNA damage response were assessed in peripheral blood mononuclear cells (PBMCs) by targeted, immuno-multiple reaction monitoring mass spectrometry (iMRM-MS) **(**Supplementary Materials page 12 and Supplementary Materials Table 3) [19]. Prior to assessing clinical trial samples, PBMCs from healthy volunteers were treated ex vivo to determine biomarkers modulated by AZD7648. Specifically, PBMC samples from healthy volunteers (*n* = 3) were treated in the presence of 1 µM AZD7648 ex vivo continuously for 4 days (Supplementary Materials page 10–11). Samples were pooled and analysed by iMRM-MS. During the clinical trial, patient blood samples were collected during cycle 1 (day 1 and day 8), cycle 2 (day 1) and at the end of treatment and were analysed by iMRM-MS [20–23]. On-treatment tumour biopsies were optional in the trial protocol and none were collected, so tumour PD studies were not conducted.

### Statistical analysis

Descriptive statistics were used for all variables, as appropriate, and calculated only when *n* ≥ 3. Where data were available for <3 patients, only minimum, maximum and number of observations were provided. Percentages were calculated using the analysis set total and for modules or doses, unless otherwise stated. Analysis sets were defined as detailed in Supplementary Materials Table 4. PFS, including median time to PFS and PFS rate at a specific timepoint, was summarized using the Kaplan–Meier method. SAS^®^ version 9.4 was used for all analyses. The PK analysis set included dosed patients for whom an adequate PK profile was obtained.

## Results

### Patients

The first patient was enrolled on 09 October 2019 and the last patient visit was completed on 07 December 2022. Thirty patients were enrolled and treated with AZD7648 alone (*n* = 14) or in combination with PLD (*n* = 16). Baseline patient and disease characteristics are shown in Table 1. Most patients were White (25/30, 83.3%) and median age was 60 years in the Monotherapy Module and 61 years in the Combination Module. All patients had received prior systemic therapy; patients treated with AZD7648 monotherapy had received more prior regimens (median 4 [1–10]) than those treated with combination therapy (median 2 [1–10]).

**Table 1:** Baseline patient and disease characteristics.

Characteristic	Monotherapy (*n* = 14)	Combination therapy (*n* = 16)
Median age, years (range)	60 (49–70)	61 (30–78)
Sex, *n* (%)		
Female	9 (64.3)	5 (31.3)
Male	5 (35.7)	11 (68.8)
Race, *n* (%)		
Black or African American	0	1 (6.3)
White	11 (78.6)	14 (87.5)
Other	3 (21.4)	1 (6.3)
Ethnicity, *n* (%)		
Hispanic or Latino	2 (14.3)	0
Not Hispanic or Latino	12 (85.7)	16 (100)
ECOG PS, *n* (%)		
0	5 (35.7)	5 (31.3)
1	9 (64.3)	10 (62.5)
Missing	0	1 (6.3)
Primary tumour location, *n* (%)		
Endometrial	0	2 (12.5)
Liver	2 (14.3)	0
Lung	0	5 (31.3)
Ovary	2 (14.3)	1 (6.3)
Pancreas	2 (14.3)	0
Uterus	0	3 (18.8)
Other^†^	8 (57.1)	5 (31.3)
Overall disease classification, *n* (%)		
Metastatic	10 (71.4)	12 (75.0)
Locally advanced	1 (7.1)	1 (6.3)
Both	3 (21.4)	3 (18.8)
Type of prior systemic therapy, *n* (%)		
Cytotoxic chemotherapy	6 (42.9)	11 (68.9)
Hormonal therapy	1 (7.1)	1 (6.3)
Immunotherapy	8 (57.1)	5 (31.3)
Targeted therapy	4 (28.6)	6 (37.5)
Other^††^	3 (21.4)	2 (12.5)
Median number of prior systemic therapies, *n* (range)	4 (1–10)	2 (1–10)

*ECOG PS* Eastern Cooperative Oncology Group performance status.

^†^Primary tumour locations are as reported by the investigator. Tumors listed as ‘Other’ are as follows: Monotherapy Module – colon, anterior chamber of eye, fallopian tube, peritoneum, pleura, prostate, rectum and small intestine (all *n* = 1); Combination Module – bladder, cervix, left gluteus maximus, lymph node, and sacrum (all *n* = 1).

^††^Other systemic therapies used included MTL-CEBPA, sipuleucel-T and chemoembolization in the Monotherapy Module and BA3021 and ABI-009 in the Combination Module.

All 14 patients who received AZD7648 monotherapy had discontinued treatment at study end; the most frequent reason was progressive disease (*n* = 9, 64.3%). One patient discontinued due to a non-AZD7648-related treatment-emergent adverse event (TEAE) (Enterococcus sepsis), one due to a serious TEAE of dyspnoea, headache and pruritus, and three (28.6%) based on investigator decision (see Supplementary Materials page 8 for full withdrawal criteria).

All 16 patients who received combination therapy had discontinued treatment with both AZD7648 and PLD by study end. The most frequent reason for discontinuation of AZD7648 was RECIST progressive disease (*n* = 7, 43.8%); 6 patients (37.5%) discontinued based on investigator decision (see Supplementary Materials page 9 for full withdrawal criteria) and three (18.8%) discontinued due to other reasons (two patients due to an infusion-related reaction, one of them at cycle 1, day 1, and one due to investigator decision). PLD was discontinued due to RECIST progressive disease (*n* = 8), meeting other protocol-specified withdrawal criteria (*n* = 3; one patient due to PLD infusion reaction at Cycle 1 Day 1, one due to investigator decision, and one due to previous toxicity with the combination of the two drugs). Two (12.5%) of 16 patients completed all treatment until disease progression and underwent all protocol-specified assessments.

No patients discontinued due to COVID-19.

### Dose escalation

In the Monotherapy Module, dosing started at AZD7648 5 mg QD. Patients subsequently received AZD7648 BID at doses of 5, 10, 20, 40, 80, and 160 mg (Fig. 1). In the Combination Module, the SRC approved AZD7648 20 mg BID for use in combination with PLD 40 mg/m^2^, but this was changed to AZD7648 20 mg QD for the first 7 days of each 28-day cycle after the first two patients in the first cohort experienced DLTs; the two additional patients in this cohort were treated with AZD7648 20 mg QD for 7 days + PLD 40 mg/m^2^. In Cohort 2, patients received AZD7648 20 mg QD for 7 days + PLD 40 mg/m^2^, i.e. the dose used for the second two patients enrolled in Cohort 1. Patients in Cohort 3 received AZD7648 30 mg QD for 7 days + PLD 40 mg/m^2^.

### Safety

In the Monotherapy Module, all patients received at least one cycle of AZD7648 and were evaluable for safety; one patient (AZD7648 5 mg once daily) completed eight cycles. The mean total treatment duration was 2.9 months. Six patients (42.9%) experienced adverse events (AEs) leading to AZD7648 interruptions and two patients (14.3%) experienced AEs leading to AZD7648 dose reduction (Table 2 and Supplementary Materials Table 5). Twelve patients (85.7%) experienced TEAEs (Table 2). The incidence of TEAEs increased with AZD7648 dose, and AZD7648 dose reductions, interruptions and discontinuations generally occurred at doses greater than 80 mg BID (Supplementary Materials Table 5). SAEs were reported in one patient receiving AZD7648 40 mg BID (grade 3 back pain and grade 3 urosepsis) and four patients receiving AZD7648 80 mg BID (grade 3 infection, grade 3 dyspnoea, headache and pruritis, grade 3 biliary infection, grade 5 COVID-19 and enterococcal sepsis). The SAE of grade 3 dyspnoea, headache and pruritus was considered possibly related to treatment; the other SAEs were not related to treatment.

**Table 2:** Safety.

Event	AZD7648 (*n* = 14)		AZD7648 + PLD (*n* = 16)	
Any AE, *n* (%)	12 (85.7)		15 (93.8)	
Any grade 3/4 AE, *n* (%)	8 (57.1)		9 (56.3)	
Any SAE, *n* (%)	5 (35.7)		6 (37.5)	
Any DLT, *n* (%)	1 (7.1)		3 (18.8)	
Death, *n* (%)	4 (28.6)		1 (6.3)	
Any treatment-related AE, *n* (%)				
Related to AZD7648	5 (35.7)		15 (93.8)	
Related to PLD	–		12 (75.0)	
Any treatment-related grade 3/4 AE, *n* (%)				
Related to AZD7648	4 (28.6)		8 (50.0)	
Related to PLD	–		5 (31.3)	
Any treatment-related SAE, *n* (%)				
Related to AZD7648	1 (7.1)		3 (18.8)	
Related to PLD	–		1 (6.3)	
Any AE leading to AZD7648 discontinuation, *n* (%)	3 (21.4)		3 (18.8)	
Any AE leading to PLD discontinuation, *n* (%)	–		3 (18.8)	
Any AE leading to AZD7648 dose modification,* *n* (%)	6 (42.9)		10 (62.5)	
Any AE leading to PLD dose modification,* *n* (%)	–		9 (56.3)	
**TEAEs occurring in** ≥**20% of patients treated with either AZD7648 monotherapy or in combination with PLD**^**†**^	**Any grade**	**Grade** ≥**3**	**Any grade**	**Grade** ≥**3**
Anaemia	4 (28.6)	1 (7.1)	11 (68.8)	4 (25.0)
Diarrhoea	4 (28.6)	1 (7.1)	2 (12.5)	0
Nausea	4 (28.6)	0	7 (43.8)	0
Vomiting	4 (28.6)	0	1 (6.3)	0
Decreased appetite	3 (21.4)	0	4 (25.0)	0
Abdominal pain	3 (21.4)	0	1 (6.3)	0
Fatigue	3 (21.4)	1 (7.1)	8 (50.0)	1 (6.3)
Urinary tract infection	2 (14.3)	0	4 (25.0)	0
Neutropenia	2 (14.3)	1 (7.1)	5 (31.3)	2 (12.5)
Constipation	1 (7.1)	0	4 (25.0)	0
Stomatitis	1 (7.1)	0	8 (50.0)	2 (12.5)
Neutrophil count decrease	NR	NR	4 (25.0)	4 (25.0)
Platelet count decrease	NR	NR	4 (25.0)	1 (6.3)
Infusion-related reaction	NR	NR	4 (25.0)	0

*AE* adverse event, *DLT* dose-limiting toxicity, *NR* not reported, *PLD* pegylated liposomal doxorubicin, *SAE* serious adverse event, *TEAE* treatment-emergent adverse event.

*Dose increased, dose reduced, or drug interrupted.

^†^TEAEs were summarised by MedDRA version 25.1.

The most frequently reported class of AEs with AZD7648 monotherapy were gastrointestinal (GI) disorders (nine patients [64.3%]), including diarrhoea, nausea, anaemia and vomiting (*n* = 4 each, 28.6%) (Table 2). Anaemia also occurred in four patients. Eight patients had grade ≥3 events, four of which were possibly related to AZD7648. One DLT was reported in a patient receiving AZD7648 160 mg BID: grade 4 increased alanine aminotransferase (ALT) and aspartate aminotransferase (AST).

In the Combination Module, all patients received at least one cycle of AZD7648 and were evaluable for safety; 4 patients completed six cycles of AZD7648 and PLD. The mean total treatment duration for AZD7648 was 3.42 months. Two patients experienced TEAEs leading to AZD7648 dose reductions, and three patients reported TEAEs leading to dose interruptions (Table 2 and Supplementary Materials Table 6**)**. Fifteen patients treated with AZD7648 combined with PLD (93.8%) experienced TEAEs, and all 15 had AEs possibly related to AZD7648 whereas 12 (75.0%) had AEs possibly related to PLD (Table 2). The incidence of TEAEs was similar in all cohorts, but the incidence of TEAEs leading to dose interruptions and modifications was higher with AZD7648 30 mg QD and PLD 40 mg/m^2^ than with other dose regimens (Supplementary Materials Table 6). SAEs were reported in six patients, of which three were considered related to AZD7648 and one related to PLD. Unrelated SAEs included: one patient with grade 3 femoral fracture and grade 3 bacterial arthritis (AZD7648 20 mg QD 7 days + PLD 40 mg/m^2^); one patient with grade 3 COVID-19 (AZD7648 30 mg QD 7 days + PLD 40 mg/m^2^); and one patient with grade 3 hip fracture, grade 4 cardiac arrest and grade 5 embolism (AZD7648 30 mg QD days + PLD 40 mg/m^2^). SAEs considered related to AZD7648 were grade 3 biliary obstruction and grade 4 decreased neutrophil count (both patients treated with AZD7648 30 mg QD 7 days + PLD 40 mg/m^2^). One SAE was deemed related to AZD7648 and PLD: grade 4 stomatitis (AZD7648 20 mg BID 28 days + PLD 40 mg/m^2^). As with AZD7648 monotherapy, the most frequently reported class of TEAEs with combination therapy was GI disorders (13 patients [81.3%]). However, 11 (68.8%) patients had anaemia, eight had fatigue (50%) and eight had stomatitis (50%) (Table 2). The most commonly reported grade ≥3 events included decreased neutrophil count (*n* = 4, 25.0%), neutropenia, anaemia and stomatitis (*n* = 2 each, 12.5%). Three DLTs were experienced by 2 patients treated with AZD7648 20 mg BID 28 days + PLD 40 mg/m^2^ (grade 3 neutropenia and grade 4 neutropenia with grade 4 stomatitis) and one patient treated with AZD7648 30 mg QD 7 days + PLD 40 mg/m^2^ (grade 3 increased ALT).

### Pharmacokinetics

The geometric mean (geoMean) concentration-time profiles of AZD7648, and PLD for patients are shown in Supplementary Materials Fig. 1. Intense PK analysis on Cycle 0 Day 1 enabled the detailed determination of PK parameters. AZD7648 exposure increased in a broadly linear manner from 5 to 160 mg BID, and AZD7648 was generally rapidly absorbed with a T_max_ of ≈1–2 h (Supplementary Materials Fig. 1). Using representative monotherapy data from the AZD7648 80 mg BID cohort (highest monotherapy dose of AZD7648 at which a DLT did not occur), the following Cycle 0 Day 1 single-dose PK parameters were observed: geoMean C_max_ 2463 nmol/L (geometric CV% ±56.7); geoMean AUC_inf_ 25180 h*nmol/L ( ± 48.76); observed t_½_ 10.32 h ( ± 39.41); and geoMean CL/F 8.353 L/h ( ± 48.76). The fraction of AZD7648 excreted unchanged in urine was low and typically ranged from 5–10% of the total administered dose. Using AZD7648 30 mg QD in combination with PLD 40 mg/m^2^ as representative data, the end-of-infusion geoMean concentration for PLD was 24750 ng/mL. This result is broadly in line with expected PLD concentrations for the 40 mg/m^2^ dose level [24].

### Pharmacodynamics

**Fig. 2:**
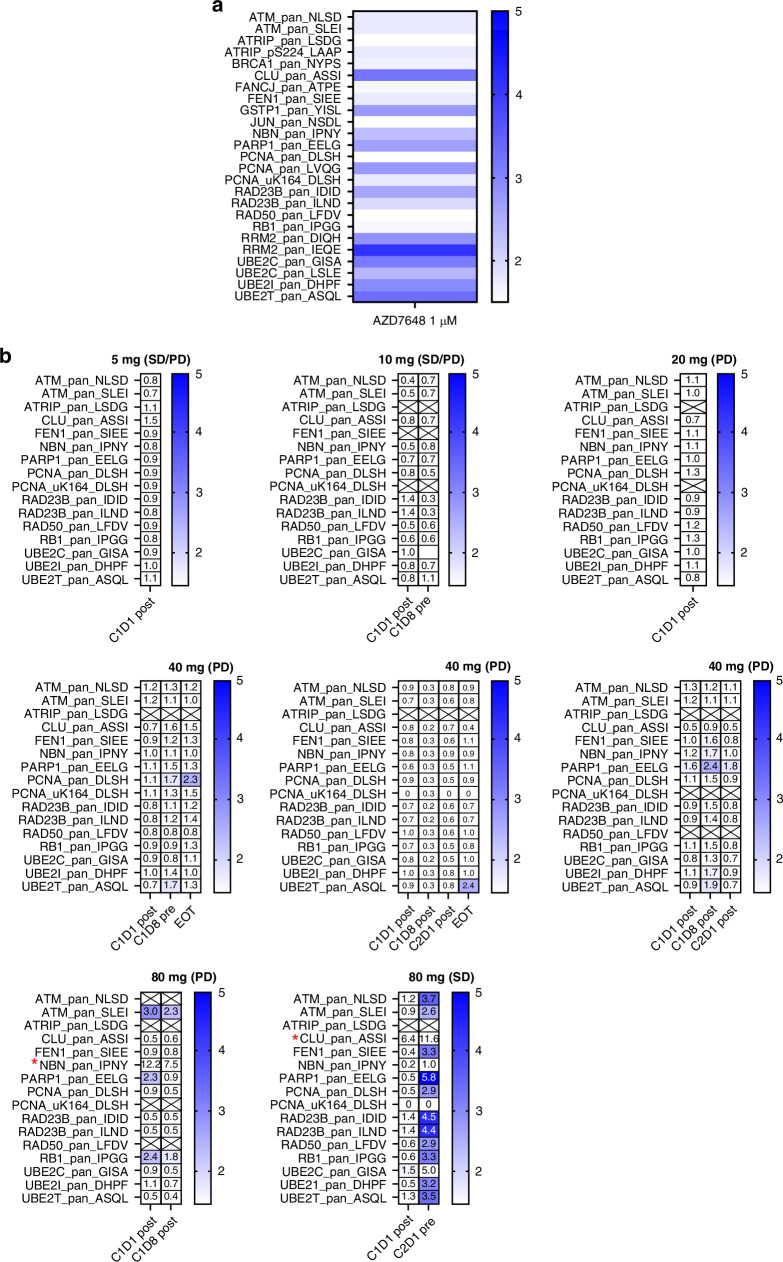
Pharmacodynamic changes in PBMCs treated with AZD7648 monotherapy. DDR protein modulation in **a** PBMCs from healthy volunteers’ treated ex vivo with AZD7648, **b** patient samples collected at different timepoints on treatment at various dose levels of AZD7648. Analytes marked with an asterisk (*) are increased above the fivefold threshold. Best RECIST response associated with observed pharmacodynamic response is shown in brackets. The healthy volunteer data were normalized to DMSO control, and analytes with >1.5-fold expression on AZD7648 are shown. The heatmaps for patient data show >1.5-fold changes compared to pre-dose (screening or C1D1 pre-dose) in selected analytes matching healthy volunteer ex vivo analysis. CXDX, Cycle X, Day X; EOT, end of treatment.

Targeted DDR protein mass spectrometry was performed on PBMCs from healthy volunteers treated ex vivo with vehicle (DMSO) or AZD7648 to identify potential PD biomarkers modulated by a DNA-PK inhibitor. The ex vivo data revealed upregulation of various proteins associated with replication, the Fanconi anaemia DNA repair pathway or homologous recombination repair, which are suggestive of engagement of alternative DDR pathways in the absence of the NHEJ mechanism (Fig. 2a and Supplementary Materials Table 7).

We then tested whether these biomarkers were detected in PBMCs collected from patients post-treatment with AZD7648 monotherapy, as potential indicators of the in vivo biological activity of the DNA-PK inhibitor. We analysed PBMCs isolated from blood samples from patients treated with AZD7648 at doses of 5 mg to 80 mg. Analysis of patient samples at different timepoints in the treatment cycle did not show any activity of AZD7648 at doses up to 40 mg. We identified one patient at the 80 mg dose level who achieved PD drug exposures at which preclinical changes had been detected (i.e. >1 µM), demonstrating induction of protein markers similar to those identified ex vivo (Fig. 2b, Supplementary Materials Table 8 and Supplementary Materials Fig. 2). However, in the absence of more samples from this dose level or higher, we cannot conclude with confidence that the observed signal is indeed associated with AZD7648 biological activity in human blood.

### Efficacy

**Fig. 3:**
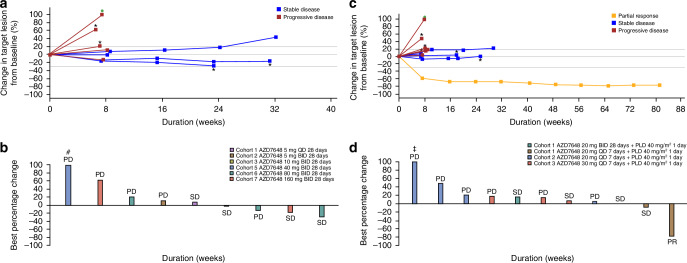
Changes in target lesion size. **a** Best percentage change in target lesion size over time and **b** waterfall plot showing best percentage change from baseline in patients treated with AZD7648 monotherapy. **c** Best percentage change in target lesion size over time and **d** waterfall plot showing best percentage change from baseline in patients treated with AZD7648 and PLD. **a** *New lesion. The reference line at +20% represents the definition of progressive disease. The reference line at −30% represents the definition for confirmed partial response. Three patients with no post baseline assessment are excluded. **b** BID twice daily, PD best response of progressive disease, QD once daily, SD best response of stable disease. ^#^Value capped as a result of the restriction to the *Y* axis scale (−100%, +100%). Increase was 108.33%. Best percentage change in target lesion size is the maximum reduction from baseline or the minimum increase from baseline in the absence of a reduction. The reference line at +20% represents the definition of progressive disease. The reference line at −30% represents the definition for confirmed partial response. Three patients with no post baseline assessment are excluded. **c** *New lesion. The reference line at +20% represents the definition of progressive disease. The reference line at −30% represents the definition of confirmed partial response. Four patients with no post baseline assessment are excluded. **d** Four patients with no post baseline assessment are excluded. ^‡^Value capped as a result of the restriction to the *Y* axis scale (−100%, +100%). Increase was 334.7%. BID twice daily, PD best response of progressive disease, PR best response of partial response, QD once daily, SD best response of stable disease.

Limited efficacy was noted with AZD7648 monotherapy within the dose ranges investigated. Of the 12 patients in the response-evaluable set, four patients (25%) had a decrease in lesion size from baseline (Fig. 3). No patient had a RECIST complete or partial response. Of the 12 patients who were evaluable for objective response, four (33.3%) patients had RECIST stable disease, and five (41.7%) patients experienced RECIST disease progression. Two (16.7%) patients died due to disease progression, and one was not evaluable for RECIST assessment. Of the four patients with RECIST stable disease, one patient treated with AZD7648 80 mg BID had a percentage change in target lesion size of −29.2% from baseline (Table 3). No pharmacodynamic data were available for this patient. One patient was not evaluable due to incomplete post-baseline assessments. Median PFS was 1.91 months (80% CI 1.64–3.42). The PFS rate at 3 months was 38.46 (80% CI 21.66–55.05) and 23.08% at 6 months (80% CI 10.20–38.99).

**Table 3:** Best objective response by RECIST 1.1 in patients who received AZD7648 monotherapy or AZD7648 in combination with PLD.

Best objective response, *n* (%)	AZD7648 5 mg QD (*n* = 1)	AZD7648 5 mg BID (*n* = 1)	AZD7648 10 mg BID (*n* = 1)	AZD7648 20 mg BID (*n* = 1)	AZD7648 40 mg BID (*n* = 2)	AZD7648 80 mg BID (*n* = 4)	AZD7648 160 mg BID (*n* = 2)	All (*n* = 12)
Complete response*	0	0	0	0	0	0	0	0
Partial response*	0	0	0	0	0	0	0	0
Stable disease^†^	1 (100.0)	0	1 (100.0)	0	0	1 (25.0)	1 (50.0)	4 (33.3)
Disease progression	0	1 (100.0)	0	1 (100.0)	1 (50.0)	1 (25.0)	1 (50.0)	5 (41.7)
Death	0	0	0	0	1 (50.0)	1 (25.0)	0	2 (16.7)
Not evaluable^‡^	0	0	0	0	0	1 (25.0)	0	1 (8.3)
**Best objective response,** ***n*** **(%)**	**AZD7648 20** **mg BID** + **PLD 40** **mg/m**^**2**^ **(*n*** = **2)**	**AZD7648 20** **mg QD 7 days** + **PLD 40** **mg/m**^**2**^ **(*n*** = **2)**		**AZD7648 20** **mg QD 7 days** + **PLD 40** **mg/m**^**2**^ **(*n*** = **5)**		**AZD7648 30** **mg QD 7 days** + **PLD 40** **mg/m**^**2**^ **(*n*** = **6)**		**All (*n*** = **15)**
Complete response*	0	0		0		0		0
Partial response*	0	1 (50.0)		0		0		1 (6.7)
Stable disease^†^	1 (50.0)	1 (50.0)		0		2 (33.3)		4 (26.7)
Disease progression	0	0		4 (80.0)		2 (33.3)		7 (46.7)
Death	0	0		0		1 (16.7)		1 (6.7)
Not evaluable^‡^	1 (50.0)	0		1 (20.0)		1 (16.7)		3 (20.0)

*BID* twice daily, *QD* once daily, *PLD* pegylated liposomal doxorubicin, *RECIST* Response Evaluation Criteria in Solid Tumours. RECIST version 1.1.

*Response required confirmation after 4 weeks.

^†^All patients with a best objective response of stable disease had stable disease duration of ≥8 weeks.

^‡^Patients who were not evaluable had incomplete post-baseline assessment.

Similarly, limited efficacy was observed with AZD7648 in combination with PLD in the dose ranges investigated (Fig. 3). One of the 15 patients in the response-evaluable set had a confirmed RECIST PR (AZD7648 20 mg QD 7 days + PLD 40 mg/m^2^), with a best change in target lesion size from baseline of −76.8% at 80 weeks. This patient had cervical adenocarcinoma pretreated with first-line carboplatin/paclitaxel/bevacizumab and second-line pembrolizumab/lenvatinib. Next generation sequencing of the tumor at baseline showed microsatellite instability high (loss of MLH1 and PMS2 and TMB 15, ARID1A G801FS*32, KRAS Q6H, PIK3CA N1044K, PTEN R130G, SMARCA4 P109FS*194 and SMARCA4 P316FS*10 mutations). Four patients had a best objective response of RECIST SD, all of ≥8 weeks in duration, seven had RECIST disease progression and three were not evaluable for RECIST due to incomplete post-baseline assessments (Table 3). Median PFS was 1.97 months (80% CI 1.81–6.11); the PFS rate at 3 months was 41.67% (80% CI 23.61–58.50) and 33.33% at 6 months (80% CI 17.05–50.54).

## Discussion

The primary objective of this study was to determine the safety and tolerability of AZD7648 alone and in combination with PLD. The design of the study ensured that dose escalation was accompanied by intensive safety monitoring and appropriate oversight by an expert SRC so that the minimum number of patients were exposed to therapy while the tolerability of the monotherapy and combination therapies was established. Overall, the SRC determined that while AZD7648 was generally well tolerated as monotherapy, dose escalation should be paused at AZD7648 160 mg BID continuous dosing and an MTD could not be declared. This was based on the observation that a lower dose of AZD7648 (20 mg) in combination with PLD, which was assessed in parallel, was not tolerated, with DLTs observed. This level of toxicity was not expected at this dose, and, overall, the combination of AZD7648 with PLD showed a high level of toxicity and a narrow therapeutic index, which led to frequent treatment discontinuations and dose modifications. Given that efficacy with AZD7648 monotherapy was not expected, further dose escalation was paused at this dose.

As noted, the most frequently reported TEAEs with AZD7648 monotherapy affected the GI and blood and lymphatic systems. Diarrhoea, nausea, vomiting and anaemia all occurred in ~30% of patients, with decreased appetite, abdominal pain and fatigue occurring in ~20% of patients. The GI TEAEs were as expected based on studies of another DNA-PK inhibitor, peposertib, in which similar GI TEAEs occurred [25–28], although the incidence of diarrhoea was possibly higher with AZD7468. AZD7648 monotherapy also caused anaemia in 28.6% of patients, with a single grade 3 event; neutropenia was also observed in 14.3% of patients. Neither anaemia nor myelosuppressive TEAEs were reported in a trial of single-agent peposertib [28], and trials of peposertib with other anticancer therapies were not randomised [25–27], meaning that whether it has any effect on anaemia or myelosuppression caused by combination agents cannot be determined.

Future development of DNA-PK inhibitors may include a requirement for primary prophylaxis with anti-emetics. In addition, to ameliorate emerging toxicities, secondary prophylaxis using G-CSF and loperamide may help with toxicities such as neutropenia and diarrhoea, respectively. Primary prophylaxis using G-CSF is not recommended based on the incidence of neutropenia when AZD7648 is used as monotherapy. However, for combinations of DNA-PK inhibitors and other modalities like radiotherapy where the incidence is likely to be higher, primary prophylaxis could be considered depending on patient-, disease- and/or treatment-related factors.

The pharmacodynamic data in PBMCs collected from a limited number of patients suggested that dose levels up to 40 mg were not biologically active and did not change DDR protein expression, as predicted from the preclinical ex vivo experiments. While there was an initial pharmacodynamic signal from a single patient receiving the 80 mg dose of AZD7648, higher dose levels would need to be further assessed to confirm target and pathway modulation. It should be noted that one of the two patients treated with AZD7648 160 mg BID experienced DLTs of grade 3 increased ALT and increased AST. Therefore, conducting future studies to explore doses of AZD7648 monotherapy above AZD7648 160 mg BID would only be justified if a predictive biomarker of AZD7648 response can be identified to enrich for tumor response to AZD7648, thus potentially improving the risk:benefit ratio.

End-of-infusion concentrations of PLD were consistent with previously published results [24]. However, additional toxicity was reported when AZD7648 was combined with PLD, with neutropenia, anaemia and leukopenia occurring more frequently with combination therapy than monotherapy. While expected, these TEAEs occurred at lower doses of AZD7648 than anticipated when combined with PLD. Initially in Cohort 1 of the Combination Module, a dose of AZD7648 20 mg was administered QD in combination with PLD 40 mg/m^2^. The two patients who received this combination dose both experienced DLTs (grade 4 neutrophil count decrease and grade 4 stomatitis; and grade 3 neutrophil count decrease). This observation led to treatment interruption and the SRC recommended that the two subsequent patients in this cohort be treated on an intermittent schedule with AZD7648 20 mg QD for 7 days of a 28-day cycle with PLD 40 mg/m^2^ every 28 days. No further DLTs were observed in these patients or in a cohort of patients treated using the same doses of both AZD7648 and PLD. However, a further DLT of grade 3 increased ALT occurred at the highest doses of the combination tested (AZD7648 30 mg QD 7 days + PLD 40 mg/m^2^). This suggested that further dose escalation of AZD7648 in combination with PLD was not possible and that the maximum dose used in this study likely did not achieve pharmacokinetic exposure levels required for combination antitumour efficacy [12].

Based on the mechanism of action of AZD7648, preclinical data and the unselected patient population, antitumor activity was not expected with monotherapy [3, 4, 12, 13]. This proved to be the case, with RECIST SD being the best response, with little or no tumour shrinkage in three of four patients with RECIST SD. When AZD7648 was given in combination with PLD, one RECIST PR and evidence of RECIST SD was observed. This level of efficacy was generally lower than that expected from AZD7648 in combination with a therapy capable of inducing DNA DSBs, such as a topoisomerase II inhibitor [12], even though such therapy is a rational combination partner for AZD7648 based on the preclinical in vivo data [12].

Of note, the one patient with a confirmed and sustained PR in the PLD combination arm was confirmed to have a cervical adenocarcinoma tumour that was mutated in both mismatch repair genes MLH1 and PMS2, with a resulting microsatellite instability high (MSI-H) phenotype. This is of interest because the ATM gene contains microsatellite sequences that are frequently associated with mononucleotide deletions in MSI-H tumors, resulting in a lack of ATM protein expression [29]. Although we are not able to confirm the loss of ATM protein in the absence of immunohistochemistry data, this provides a plausible explanation for the long-term response of this tumour, because PLD-induced DNA DSBs would not be effectively repaired in the absence of functional ATM and DNA-PK.

Expression of DNA-PK and NHEJ activity is not limited to cancer cells but is also important for DNA damage response in normal tissue, including the bone marrow. The on-target toxicities observed with AZD7648, which are further exacerbated by combination with DNA-damaging chemotherapy, may be partially managed by prophylactic use of G-CSF or anti-emetic drugs. However, and more importantly, optimal dosing strategies to minimize these toxicities while achieving therapeutic efficacy against cancer cells should be driven by understanding and exploiting biological differences between DNA damage response in normal and tumour tissues. For other DDR inhibitor combinations with chemotherapy, a gap schedule approach, where 2–3-days between the DNA-damaging agent and DDR inhibitor treatment provides time for bone marrow DNA damage to be resolved, has been described preclinically and tested clinically [30]. DNA-PK inhibitor combinations with targeted topoisomerase 2 inhibitors could also represent a better tolerated alternative, although we are not aware that any antibody-drug conjugates with topoisomerase 2 payloads are currently available. In either case, it would be of interest to explore MSI-H tumours as a potential enrichment for ATM deficiency. Other, non-PLD combinations that induce DNA DSBs could also be of interest, such as radiotherapy. However, DNA-PK inhibition has a very high dose modification ratio (DMR) [31], and as such patient-to-patient variability in DNA-PK inhibitor exposure represents a major challenge. Perhaps a more promising approach for the future would be the combination of DNA-PK inhibitors with targeted radiation, i.e. via radio-ligand therapy. Alpha-radioconjugates in particular would be of interest due to the primary form of this high linear energy transfer radiation-induced DNA damage being complex DNA DSBs where DNA-PK is likely to play an important repair role.

The toxicity observed with AZD7648 in combination with PLD was greater than expected and the antitumour activity of both AZD7648 monotherapy and the combination was limited, leading to the early termination of this study. Based on its risk:benefit ratio, the combination of AZD7648 with PLD does not warrant further investigation using concurrent scheduling.

## Supplementary information

Supplemental Material

## Data Availability

Data underlying the findings described in this article may be obtained in accordance with AstraZeneca’s data sharing policy described at https://astrazenecagrouptrials.pharmacm.com/ST/Submission/Disclosure. Data for studies directly listed on Vivli can be requested through Vivli at www.vivli.org. Data for studies not listed on Vivli can be requested through Vivli at https://vivli.org/members/enquiries-about-studies-not-listed-on-thevivli-platform/. An AstraZeneca Vivli member page is also available outlining further details: https://vivli.org/ourmember/astrazeneca/.
