# Supplementary material for: The DNA-PK inhibitor AZD7648 alone or combined with pegylated liposomal doxorubicin in patients with advanced cancer: results of a first-in-human Phase I/IIa study
Source: Br J Cancer. 2025 May 17;133(2):168–77. doi: 10.1038/s41416-025-03053-x (PMC12304285; doi:10.1038/s41416-025-03053-x)
Supplement: Supplementary file 1 — Supplemental Material [file 41416_2025_3053_MOESM1_ESM.pdf]

# Supplementary Materials

## Inclusion Criteria

### Informed consent

- Had to be capable and willing to give signed informed consent which included compliance with the requirements and restrictions listed in the informed consent form (ICF) and in this protocol
- Had to provide a signed and dated written genetic informed consent prior to collection of samples for exploratory genetic analysis (optional)

### Age

- Patient had to be at least 18 years of age, at the time of signing the ICF

### Type of patient and disease characteristics

- Patients had to have histological or cytological confirmation of advanced malignancy considered to be suitable for study treatment
- Eastern Cooperative Oncology Group – Performance Status (ECOG-PS) of 0 to 1
- Life expectancy > 12 weeks
- Progressive cancer at the time of study entry
- Progressive disease (PD) expansion cohorts (or PD expansion subgroup): Patients had to have at least one tumour suitable for biopsy and had to consent to having biopsies collected

### Reproduction

- Negative pregnancy test (urine or serum) prior to the start of dosing for women of child-bearing potential. Women of child-bearing potential were defined as women between menarche and menopause who have not been permanently or surgically sterilised and were not capable of procreation
- Female patients had to be post-menopausal, surgically sterile, or using an acceptable method of contraception for the duration of the study (from the time they sign consent) and for 12 weeks after the last dose of study treatment to prevent pregnancy
- For the duration of the study (from the time they sign consent) and for 12 weeks after the last dose of study treatment, sexually active male patients had to be willing to use contraception

Post-menopausal was defined as:

- No menses for 12 months without an alternative medical cause. A high follicle stimulating hormone (FSH) level in the post-menopausal range could be used to confirm a post-menopausal state in women not using hormonal contraception or hormonal replacement therapy. However, in the absence of 12 months of amenorrhea, a single FSH measurement was insufficient
- Radiation-induced oophorectomy with last menses >12 months prior to the study
- Chemotherapy-induced menopause with >12-month interval since last menses
- Surgical sterilisation (bilateral oophorectomy or hysterectomy)

Post-menopausal was defined as:

- No menses for 12 months without an alternative medical cause. A high FSH level in the
- post-menopausal range could be used to confirm a post-menopausal state in women not using hormonal contraception or hormonal replacement therapy. However, in the absence of 12 months of amenorrhea, a single FSH measurement was insufficient
- Radiation-induced oophorectomy with last menses >12 months prior to the study
- Chemotherapy-induced menopause with >12-month interval since last menses
- Surgical sterilisation (bilateral oophorectomy or hysterectomy)

**Combination Module-specific inclusion criteria**

Patients were eligible to be included in Combination Module 1 of the study only if all the inclusion criteria applied.

- Patients had to meet the eligibility criteria described in the Core Module
- Suitable for treatment with pegylated liposomal doxorubicin (PLD) as per local prescribing information
- Left ventricular ejection fraction above the institutional lower limit of normal, as assessed by echocardiography or multigated acquisition (MUGA) scan

## Exclusion criteria

The exclusion criteria that were applicable to all parts/cohorts of the study are described in this section.

**Medical conditions**

- Any unresolved toxicities from prior therapy CTCAE Grade ≥ 2 (with the exception of alopecia)

- Spinal cord compression or brain metastases unless definitively treated (minimum of three weeks between completion of radiotherapy and first dose of study treatment and recovery from acute toxicity Grade ≥2), asymptomatic, stable (no clinical evidence of
- progression since completion of central nervous system-directed therapy) and not requiring steroids for at least four weeks. Disease outside the central nervous system had to be present
- As judged by the investigator, any evidence of severe or uncontrolled medical conditions including but not limited to:
  - Uncontrolled diabetes mellitus, uncontrolled seizures, active infection requiring systemic antibiotics, antifungal or antiviral drugs, severe chronic obstructive pulmonary disease, severe Parkinson's disease, active inflammatory bowel disease, psychiatric condition, active bleeding diatheses, renal transplant, or active infection including any patient with active hepatitis B, hepatitis C or human immunodeficiency virus
- Any other malignancy which had been active or treated within the past three years, with the exception of in situ cancer of the cervix, non-melanoma skin cancer, ductal carcinoma in situ, Stage 1 Grade 1 endometrial carcinoma, or other solid tumours including lymphomas (without bone marrow involvement) curatively treated with no evidence of disease for ≥5 years
- Refractory nausea and vomiting or unable to swallow and retain oral medication, chronic gastrointestinal (GI) diseases or previous bowel resection with clinically significant sequelae that could preclude adequate absorption of AZD7648, GI symptoms common terminology criteria for adverse events (CTCAE) Grade >1, history of GI ulceration and gastrointestinal haemorrhage within six months of first study treatment administration

**Prior/concomitant therapy**

- Were receiving or have received anti-cancer treatment within the following periods prior to the first dose of investigational product:
  - Cytotoxic treatment: three weeks
  - Non-cytotoxic drugs, including small molecule investigational products: three weeks or five half-lives (whichever is longest)
  - Biological products including investigational immuno-oncology agents: four weeks
  - Radiation with a limited field for palliation: one week (three months for radiation to the abdomen or pelvis)
  - Radiation to > 30% of the bone marrow or with a wide field: four weeks
  - Lung radiation: 60 days
  - Major surgery: four weeks; minor surgery or biopsy: one week

- Received corticosteroids at a dose of ≥10 mg prednisone/day or equivalent for any reason during the four weeks prior to the first dose. Ongoing low dose steroids for longer than three months (excluding inhalational, nasal, creams, lotions, and gels) were not allowed
- Receiving or having received concomitant medications, herbal supplements and/or foods known to significantly modulate CYP3A4 activity (potent/strong inhibitors or inducers of CYP3A4). The required washout period prior to starting study treatment was three weeks (five weeks for enzalutamide or phenobarbital) until 28 days after the last dose of study treatment. Patients could receive a stable dose of bisphosphonates or denosumab for bone metastases, before and during the study if these were started at least two weeks prior to study treatment

**Prior/concurrent clinical study experience**

- Prior exposure to a DNA-dependent protein kinase (DNA-PK) inhibitor or hypersensitivity to any excipient of the product
- Cardiac dysfunction as defined by any of the following within 6 months of study entry:
  - Acute myocardial infarction.
  - New York Heart Association Class II/III/IV heart failure
  - Unstable angina
  - Unstable cardiac arrhythmias e.g., clinically important abnormalities in conduction or morphology of resting ECG such as complete left bundle branch block or third-degree-heart block
- Any of the following cardiac criteria:
  - Known reduced left ventricular ejection fraction below the institutional lower limit of normal (LLN)
  - Mean resting QTc >470 milliseconds obtained from three echocardiograms (ECGs) in 24 hours using the Fridericia formula
  - Any factors that increased the risk of QTc prolongation or arrhythmic events such as hypokalaemia, congenital long QT syndrome, immediate family history of long QT syndrome or unexplained sudden death under 40 years of age
- Inadequate haematological or organ function as defined by:
  - Haemoglobin <90 g/L with no blood transfusions or erythropoietin within 14 days of obtaining these values or before starting treatment
  - Absolute neutrophil count <1500 cells/mm3 (<1.5 x 109 /L) with no haematopoietic growth factors within 14 days of obtaining these values or before starting treatment

- Platelet count < 100,000/mm3 (<100 x 109/L) with no platelet transfusions within14 days of obtaining these values or before starting treatment international normalised ratio (INR) ≥1.5 or other evidence of impaired hepatic synthesis function
- Bilirubin ≥1.5 x upper limit of normal (ULN) or ≥2 x ULN for patients with documented/suspected Gilbert's disease (or likely to be in three weeks)
- AST or ALT ≥2.5 x ULN if no demonstrable liver metastases or ≥5 x ULN in the presence of liver metastases (or likely to be in 3 weeks)
- Creatinine clearance <50 mL/minute, as assessed using Cockcroft-Gault, EDTA clearance or 24 hours urine collection

**Other exclusions**

- Involvement in the planning and/or conduct of the study (applies to both AstraZeneca staff and/or staff at the study site)
- Judgement by the investigator that the patient could not participate in the study if the patient was unlikely to comply with study procedures, restrictions, and requirements
- Previous enrolment in the present study
- For female patients only: currently pregnant (confirmed with positive pregnancy test) or breast-feeding
- For host genetics research study (optional):
  - Previous allogenic bone marrow transplant
  - Non-leukocyte depleted whole blood transfusion within 120 days for the date of the genetic sample collection
- For food effect cohort only: insulin-dependent diabetes
- History and/or presence of COVID-19:
  - Previous severe course of COVID-19 (i.e., hospitalisation, extracorporeal membrane oxygenation, mechanically ventilated)
  - Clinical signs and symptoms consistent with COVID-19, e.g., fever, dry cough, dyspnoea, sore throat, fatigue or confirmed current infection by appropriate laboratory test within the last 4 weeks prior to screening

**Combination Module-specific exclusion criteria**

Patients could not enter Combination Module 1 of the study if any of the exclusion criteria applied.

- Any contraindication to the PLD or any excipients as per local prescribing information e.g., hypersensitivity
- Patients who had drainage of their ascites during the preceding 4 weeks prior to enrolment of the study (patients with indwelling peritoneal catheters were permitted)

- Patients who had a previous CTCAE Grade ≥ 4 haematological toxicity with PLD

## Definitions of dose-limiting toxicity

DLTs were evaluated during Cycle 0 and Cycle 1 of treatment. Toxicity was graded according to the National Cancer Institute CTCAE v5.0.

A DLT was defined as an AE that occurs from the first dose of study treatment up to and including Cycle 1, Day 28 (the DLT assessment period) that is assessed as unrelated to the disease, intercurrent illness, or concomitant medications and that, despite optimal therapeutic interventions, meets any of the following criteria:

Haematological Toxicities:

- Grade 4 neutropenia lasting >4 days.
- Grade 4 thrombocytopenia.
- Grade 3 neutropenia (ANC ≥500 to <1000 cells/mm$^3$) of any duration accompanied by fever ≥38.5ºC and/or systemic infection.
- Grade 3 thrombocytopenia (25,000 to <50,000/mm$^3$) with bleeding

Non-haematological toxicities:

- Confirmed laboratory abnormalities
- Nausea or vomiting for more than three consecutive days despite administration of maximal anti-emetic therapy
- Diarrhoea for more than three consecutive days despite administration of maximal anti-diarrheal therapy
- QTc prolongation >500 milliseconds or QTcF prolongation from baseline by 60 milliseconds confirmed on at least two separate ECGs
- Any other toxicity that is greater than at baseline AND is clinically significant and/or unacceptable, and does not respond to optimal therapeutic intervention within 72 hours AND is judged to be a DLT by the SRC
- Any treatment-related event, including significant dose reductions, omissions or delays, judged to be a DLT by the SRC. Examples may include CTCAE Grade 2 toxicities that are clinically significant and/or unacceptable according to the investigator, toxicities that result in an inability to administer at least 75% of study treatment during Cycle 1 or delay the administration of study treatment in the subsequent cycle by ≥7 consecutive days

DLT will not include the following:

- Transient isolated laboratory abnormalities which are not considered clinically significant and resolve to baseline within 72 hours without any intervention
- Alopecia
- Toxicity unrelated to treatment e.g., related to the underlying disease or disease-related

- process under investigation
- Grade 4 vomiting and diarrhoea lasting <72 hours in the absence of maximal medical therapy
- Grade 3 nausea, vomiting or diarrhoea that lasts <48 hours and resolves to Grade ≤1 either spontaneously or with maximal medical therapy
- Grade 3 fatigue <5 days
- Grade 3 hypertension in the absence of maximal medical therapy
- Grade 3 electrolyte abnormalities that resolve to Grade ≤1 within 48 hours spontaneously or with conventional medical intervention and is not clinically complicated
- Grade 3 rash that resolves to Grade ≤1 within three weeks
- Grade 3 or Grade 4 elevation in serum amylase and/or lipase that are not associated with clinical or radiographic evidence of pancreatitis

## Withdrawal criteria

### Discontinuation of study treatment

Patients may be discontinued from study treatment in the following situations. Note that discontinuation from study treatment is NOT the same thing as a complete withdrawal from the study:

- Disease progression (confirmed progression or symptomatic deterioration or confirmed progression by RECIST criteria)
- AE, e.g., study treatment-related toxicity that fail to recover to CTCAE Grade ≤2 or the patient's baseline within 28 days. Patients that withdraw due to treatment-related toxicity must be observed until resolution of toxicity to CTCAE Grade ≤1 or the patient's baseline
- Patient or Investigator decision. The patient is at any time free to discontinue treatment, without prejudice to further treatment
- Pregnancy
- Non-compliance with the Clinical Study Protocol (CSP) (Investigator or patient)
- Patients incorrectly initiated on study treatment
- Unexpected, significant or unacceptable risk to the patients enrolled in the study
- Lack of evaluable and/or complete data
- Decision to modify the development plan of the drug
- Sponsor termination of study due to unfavourable risk-benefit

## Characterisation of proteomic changes induced by AZD7648 in healthy volunteers' PBMCs

PBMCs were isolated from leukocyte cones by density gradient centrifugation (Lymphoprep, STEMCELL technologies). Leukocyte cones were commercially acquired and supplied by NHS Blood and Transplant Service (NHSBT, Cambridge, UK) as anonymized samples from consenting donors. AstraZeneca has a governance framework and processes in place to ensure that commercial sources have appropriate patient consent and ethical approval in place for collection of the samples for research purposes including use by for-profit companies. The AstraZeneca Biobank in the UK is licensed by the Human Tissue Authority (Licence No. 12109) and has National Research Ethics Service Committee (NREC) Approval as a Research Tissue Bank (RTB) (REC No 17/NW/0207) which covers the use of the samples for this project.

PBMCs were isolated as previously described.[1] Briefly, leukocyte cones resulting from plateletpheresis were diluted with four volumes of PBS, layered over a 0.75 volumes of Lymphoprep, centrifuged at 800 x g for 30 minutes with no brake. PBMCs were harvested from the Lymphoprep-plasma interface and re-suspended in RPMI medium (SIGMA R8758-500ML) supplemented with 10% heat-inactivated FBS (Hyclone #SH30071.03HI), 100 units/mL of penicillin, 100 units/mL streptomycin (Gibco #15070-063), 2mM L-Glutamine (Gibco #25030-081). Diluted PBMC preparation was spun down and treated with Red Blood Cell Lysis Buffer (Gibco # A10492-01) for 3 minutes, washed and re-suspended in supplemented RPMI medium. PBMCs were cultured in supplemented RPMI medium at 3 x 106 cells/mL for 4 days at 37oC and 5% CO2 in T175 flasks with fresh growth medium in presence of DMSO or 1 µM AZD7648 and harvested on day 4. PBMC pellets were submitted for immuno-multiple reaction monitoring (iMRM) mass spectrometry analysis, as previously described.[1,2]

Disclaimer: This report is independent research. NHS Blood & Transplant have provided material in support of the research. The views expressed in this publication are those of the authors and not necessarily those of NHS Blood & Transplant.

### Reference

1. Whiteaker JR, Wang T, Zhao L, et al. Targeted mass spectrometry enables quantification of novel pharmacodynamic biomarkers of ATM kinase inhibition. Cancers (Basel) 2021;13:3843.

2. Jones GN, Rooney C, Griffin N, et al. pRAD50: a novel and clinically applicable pharmacodynamic biomarker of both ATM and ATR inhibition identified using mass spectrometry and immunohistochemistry Br J Cancer 2018;119:1233–1243.

**Preparation of Clinical PBMCs and iMRM analysis**

Blood from patients was drawn into 8 mL Vacutainer CPT tubes (BD) before treatment, 2-8h post dose at C1D1, C1D8, C2D1 and at discontinuation.

After inverting the tube 8-10 times, it was centrifuged and the PBMC layer transferred into a new tube, washed once with ice cold PBS, centrifuged again to obtain a pellet which was then snap frozen.

PBMC pellets were lysed with 8M Urea supplemented with proteases and phosphatases inhibitors, sonicated, clarified by centrifugation and quantified with Micro-BCA assay (Thermo-Scientific).

The samples were quality checked by SDS-PAGE followed by silver staining to evaluate the complexity of the obtained protein extract and assess the presence of most common blood contaminants (hemoglobulin and albumin). Out of 55 PBMC pellets received, 35 met the criteria for analysis (protein extract ≥ 200ug and sufficient bands complexity in SDS page).

The protein extract of each sample was combined with the mix of heavy stable isotope peptides of the DDR1 and DDR2 panel (**Supplementary Table 3**) plus the QC proteins extract, proteolyzed with Lys-C/Trypsin, desalted and then subjected to the iMRM workflow [1].

Samples were run on a 5500 QTRAP mass spectrometer (Sciex) in line with a Eksigent 425 LC system, nanoflex using a 0.075 x 150 mm column (Reprosil/ChromXP C18 45oC). Samples were trapped and eluted at 300 nL/min.

Raw data have been inspected for consistency between the retention time of the heavy standard versus the endogenous peptide; the signal ratio between heavy vs light peptide has been reported for each analyte being greater than LOQ.

To interpret the data, signals have been normalised to account for equal protein loading. Additionally, data were normalised to CD45 level of expression, a PBMC specific marker, to correct for variable sample purity (e.g. due to red blood cells presence).

Data for each patient are shown as fold-change at individual timepoints compared to the pre-dose value.

**Reference**


1. Whiteaker JR, Zhao L, Saul R, et al. A multiplexed mass spectrometry-based assay for robust quantification of phospho signaling in response to DNA damage. Radiat Res 2018;189:505–518.

**Supplementary Table 1. AZD7648 PK sample collection schedule (AZD7648 monotherapy)**

| | Blood | Urine |
|---|---|---|
| Cycle 0, Day 1 | Pre-dose and 15 minutes (minimum), 30 minutes, 60 minutes, 2 hours, 4 hours, 8 hours, 10 hours and 12 hours post-dose | Pre-dose and 0–8 hours and 8–24 hours post-dose |
| Cycle 0, Day 2 | 24 hours post-dose | |
| Cycle 0, Day 3 | 48 hours post-dose | |
| Cycle 0, Day 4 | 72 hours post-dose | |
| Cycle 1, Day 1 | Pre-dose | |
| Cycle 1, Day 8 (or visit Y* for intermittent schedules) | Pre-dose and 15 minutes (minimum), 30 minutes, 60 minutes, 2 hours, 4 hours, 8 hours, 10 hours and 12 hours post-dose | 0–8 hours and 8–24 hours post-dose; urine not required at visit Y* |
| Cycle 2, Day 1 | Pre-dose | |
| Cycle 3, Day 1 | Pre-dose | |
| Cycle X, Day 1 (if a patient has a dose escalation to another dose, then, at the time of escalation); this provides the starting exposure at the time of escalation | Pre-dose and 1 hour post-dose | |
| Cycle X+1, Day 1 (if a patient has a dose escalation at Cycle X then, at the next cycle Day 1) | Pre-dose and 1 hour post-dose | |
| Discontinuation | Any sample between 0 and 72 hours post-last dose | |

*Visit Y represents the last AZD7648 dosing day in the first block of AZD7648 treatment in a cycle, e.g., if AZD7648 is dosed on Days 1 to 7, Visit Y is Day 7; if AZD7648 is dosed on Days 1 to 3, Visit Y is Day 3.

**Supplementary Table 2. AZD7648 PK sample collection schedule (AZD7648 + PLD)**

| | Blood for AZD7648 | Blood for PLD |
|---|---|---|
| Cycle 0, Day 1 | Pre-dose and 15 minutes (minimum), 30 minutes, 60 minutes, 2 hours, 4 hours, 6 hours and 8 hours post-dose | |
| Cycle 0, Day 2 | 24 hours post-dose | |
| Cycle 0, Day 3 | 48 hours post-dose | |
| Cycle 0, Day 4 | 72 hours post-dose | |
| Cycle 1, Day 1 | Pre-dose and 2 hours post-dose | Pre-dose and 4 hours after start of infusion |
| Cycle 1, Day 8 (or visit Y* for intermittent schedules) | Pre-dose and 15 minutes (minimum), 30 minutes, 60 minutes, 2 hours, 4 hours, 6 hours and 8 hours post-dose | |
| Cycle 2, Day 1 | Pre-dose | Pre-dose and 4 hours after start of infusion |
| Cycle 3, Day 1 | Pre-dose | |
| Discontinuation | Any sample between 0 and 72 hours post-last dose | |

*Visit Y represents the last AZD7648 dosing day in the first block of AZD7648 treatment in a cycle, e.g., if AZD7648 is dosed on Days 1 to 7, Visit Y is Day 7; if AZD7648 is dosed on Days 1 to 3, Visit Y is Day 3.

**Supplementary Table 3. Peptides and phosphopeptides included in immuno-MRM Mass Spectrometry panel used on healthy volunteer and clinical PBMC samples**

| Non-modified peptides | Modified peptides | Non-modified peptides | Modified peptides |
|---|---|---|---|
| UBE2C_pan | | UBE21_pan | |
| UBE2C_pan | | FAAP100_pan | FAAP100_pS667 |
| CHEK1_pan | CHEK1_pS317 | ATM_pan | ATM_pS2996 |
| FANCA_pan | | ATM_pan | ATM_pS367 |
| RAD51C_pan | | ATR_pan | ATR_pT1989 |
| REV3L_pan | REV3L_pS1724 | MDC1_pan | MDC1_pS329 |
| NBN_pan | NBN_pS432 | TERF2_pan | FERF2_pS323 |
| NBN_pan | NBN_pS343 | RIF1_pan | |
| JUN_pan | JUN_pS63 | | PALB2_pS376 |
| RB1_pan | RB1_pS807 | ATRIP_pan | ATRIP_pS224 |
| GSTP1_pan | | TOPBP1_pan | TOPBP1_pT1062 |
| PARP1_pan | PARP1_pS177 | TOPBP1_pan | |
| CLU_pan | | RAD50_pan | RAD50_pS635 |
| PCNA_pan | PCNA_uK164 | FANCJ_pan | FANCJ_pS1032 |
| PCNA_pan | | FANCJ_pan | FANCJ_pT918 |
| RRM2_pan | | UBE2T_pan | UBE2T_pS184 |
| RRM2_pan | | RAD18_pan | RAD18_pS471 |
| BRCA1_pan | BRCA1_pS1524 | | RAD18_pS99 |
| FEN1_pan | | | FANCI_pS730 |
| BRCA2_pan | BRCA2_pS1680 | FANCD2_pan | FANCD2_uK561 |
| RAD23B_pan | | FANCD2_pan | H2AX_pS139 |
| RAD23B_pan | | | |
| CHEK1_pan | CHEK1_pS286 | SAAL1_pan | SAAL1_pS237 |
| | LAT_pS224 | UTP14A_pan | UTP14A_pS453 |
| PAK4_pan | PAK4_s181 | POLQ_pan | |
| | | | RAD50_pS470 |
| GAPDH_pan | | | LMNB1_pT20; pS23 |
| | TP53_pS315 | | LMNB1_pT20 |
| | TP53_pS15 | | |
| CDK1_pan | CDK1_pT161 | | LMNB1_pS23 |
| CDC25B_pan | CDC25B_pS323 | | CDC25B_pS160 |
| CASP3_pS26 | | LIME1_pan | LIME1_pT274 |

| Non-modified peptides | Modified peptides |
|---|---|
| MKI67_pan | MKI67_pT1801 |
| | MKI67_pT2406 |
| | CDK7_pT170 |
| | |
| TNFRSF17_pan | TNFRSF17_pS173 |
| MCM6_pan | MCM6_pS762 |
| | NUMA1_pS395 |
| | CDCA8_pT106 |
| | NCAPH2_pS492 |
| CASC5_pan | CACS_pS767 |
| RTF1_pan | RTF1_pS655 |

| Non-modified peptides | Modified peptides |
|---|---|
| | |
| CDC25C_pan | CDC25C_pS216 |
| CHEK2_pan | CHEK2_pT387 |
| | CHEK2_pS379 |
| MDM2_pan | MDM2_pS166 |
| MRE11A_pan | MRE11A_pS676 |
| | PARP1_pS41 |
| RAD9A_pan | RAD9A_pS387 |
| TP53BP1_pan | TP53BP1_pT543; pS552 |
| | TP53BP1_pT543 |
| | TP53BP1_pS552 |
| ACT_pan | |
| TUBB_pan | |

**Supplementary Table 4. Analysis sets**

| Analysis set | Definition |
|---|---|
| Enrolled set | All patients |
| Safety set | All patients who received ≥1 dose of study treatment |
| DLT evaluable set | All patients who received at least 1 dose of any study treatment and either experienced DLT during Cycle 0 or 1, or who completed minimum safety evaluation requirements and received at least 75% of the total amount of planned dose of AZD 7648 (and PLD for Combination Module 1). In the event of an intra-patient dose escalation, such patients will not be evaluable for DLT at the escalated dose. |
| PK set | All patients who received at least 1 dose of any study treatment and had at least 1 reportable post first dose concentration without any protocol deviations that might have affected PK. |
| PD set | All patients who received at least 1 dose of any study treatment with at least 1 reportable PD measurement. |
| Evaluable for efficacy set | All patients who received at least 1 dose of any study treatment and had a baseline tumour assessment according to RECIST 1.1. |
| Evaluable for objective response | All patients who had a measurable baseline disease* by RECIST 1.1 assessment and received at least 1 dose of any study treatment. |

DLT: dose-limiting toxicity; PD: pharmacodynamics; PK: pharmacokinetics; PLD: pegylated liposomal doxorubicin; RECIST: Response Evaluation Criteria in Solid Tumours.

*Measurable disease was defined as having at least 1 measurable target lesion not previously irradiated, which was ≥10 mm in the longest diameter (except lymph nodes, which had to have a short axis ≥15 mm).

**Supplementary Table 5. Safety of AZD7648 monotherapy in the Monotherapy Module**

| | AZD7648 5 mg QD (n=1) | AZD7648 5 mg BID (n=1) | AZD7648 10 mg BID (n=1) | AZD7648 20 mg BID (n=1) | AZD7648 40 mg BID (n=3) | AZD7648 80 mg BID (n=4) | AZD7648 160 mg BID (n=3) |
|---|---|---|---|---|---|---|---|
| Any AE, n (%) | 0 | 1 (100) | 1 (100) | 1 (100) | 2 (66.7) | 4 (100) | 3 (100) |
| Any grade 3/4 AE, n (%) | 0 | 0 | 0 | 0 | 2 | 4 | 2 (66.7) |
| Any SAE, n (%) | 0 | 0 | 0 | 0 | 1 (33.3) | 4 (100) | 0 |
| Any DLT, n (%) | 0 | 0 | 0 | 0 | 0 | 0 | 1 (50.0;) |
| Any AE leading to death, n (%) | 0 | 0 | 0 | 0 | 0 | 1 (25.0) | 0 |
| Any AZD7648-related AE leading to death, n (%) | 0 | 0 | 0 | 0 | 0 | 0 | 0 |
| Any AZD7648-related AE, n (%) | 0 | 0 | 0 | 0 | 1 (33.3) | 2 (50.0) | 2 (66.7) |
| Any AZD7648-related grade 3/4 AE, n (%) | 0 | 0 | 0 | 0 | 0 | 0 | 0 |
| Any AZD7648-related SAE, n (%) | 0 | 0 | 0 | 0 | 0 | 1 (25.0) | 0 |
| Any AE leading to AZD7648 discontinuation, n (%) | 0 | 0 | 0 | 0 | 0 | 3 (75.0) | 0 |
| Any AE leading to AZD7648 dose modification, n (%) | | | | | | | |
| Reduction | 0 | 0 | 0 | 0 | 0 | 1 (25.0) | 1 (33.3) |
| Interruption | 0 | 0 | 0 | 0 | 1 (33.3) | 3 (75.0) | 2 (66.7) |
| Modification* | 0 | 0 | 0 | 0 | 1 (33.3) | 3 (75.0) | 2 (66.7) |

*Dose reduced or interrupted

**Supplementary Table 6. Safety of AZD7648 combined with PLD in the Combination Module**

| | AZD7648 20 mg BID + PLD 40 mg/m$^2$ (n=2) | AZD7648 20 mg QD 7 days + PLD 40 mg/m$^2$ (n=2) | AZD7648 20 mg QD 7 days + PLD 40 mg/m$^2$ (n=5) | AZD7648 30 mg QD 7 days + PLD 40 mg/m$^2$ (n=7) |
|---|---|---|---|---|
| Any AE, n (%) | 2 (100) | 2 (100) | 4 (80.0) | 7 (100) |
| Any grade 3/4 AE, n (%) | 2 (100) | 2 (100) | 0 | 5 (71.4) |
| Any SAE, n (%) | 1 (50) | 1 (50.0) | 0 | 4 (57.1) |
| Any DLT, n (%) | 2 (100) | 0 | 0 | 1 (14.3) |
| Any AE leading to death, n (%) | 0 | 0 | 0 | 1 (14.3) |
| Any treatment-related AE, n (%) | | | | |
| AZD7648 related | 2 (100) | 2 (100) | 4 (80.0) | 7 (100) |
| PLD related | 2 (100) | 2 (100) | 4 (80.0) | 4 (57.1) |
| Any treatment-related grade 3/4 AE, n (%) | | | | |
| AZD7648 related | 2 (100) | 2 (100) | 0 | 4 (57.1) |
| PLD related | 2 (100) | 2 (100) | 0 | 1 (14.3) |
| Any treatment-related SAE, n (%) | | | | |
| AZD7648 related | 1 (50.0) | 0 | 0 | 2 (28.6) |
| PLD related | 1 (50.0) | 0 | 0 | 0 |
| Any treatment-related AE leading to death, n (%) | | | | |
| AZD7648 related | 0 | 0 | 0 | 0 |
| PLD related | 0 | 0 | 0 | 0 |

| | AZD7648 20 mg BID + PLD 40 mg/m$^2$ (n=2) | AZD7648 20 mg QD 7 days + PLD 40 mg/m$^2$ (n=2) | AZD7648 20 mg QD 7 days + PLD 40 mg/m$^2$ (n=5) | AZD7648 30 mg QD 7 days + PLD 40 mg/m$^2$ (n=7) |
|---|---|---|---|---|
| Any AE leading to treatment discontinuation, n (%) | | | | |
| AZD7648 related | 0 | 1 (50.0) | 1 (20.0) | 1 (14.3) |
| PLD related | 0 | 1 (50) | 1 (20.0) | 1 (14.3) |
| Any AE leading to AZD7648 dose modification, n (%) | | | | |
| Reduction | 1 (50.0) | 0 | 0 | 1 (14.3) |
| Interruption | 2 (100) | 2 (100) | 1 (20.0) | 4 (57.1) |
| Modification* | 2 (100) | 2 (100) | 1 (20.0) | 5 (71.4) |
| Any AE leading to PLD dose modification, n (%) | | | | |
| Reduction | 0 | 0 | 0 | 1 (14.3) |
| Interruption | 2 (100) | 1 (50.0) | 1 (20.0) | 4 (57.1) |
| Modification* | 2 (100) | 1 (50.0) | 1 (20.0) | 5 (71.4) |

*Dose reduced or interrupted.

**Supplementary Table 7. List of analytes identified in PBMCs from healthy donors treated with 1 μM AZD7648, following iMRM-MS analysis.**

The values indicated in columns LOD PAR,CTRL and 1uM AZD7648 represent the ratio between the light versus the heavy peptide. The table shows, for each analyte, the sequence of the identified peptide, the LOQ and the CV of a reference control sample run concurrently with the samples. The table shows only peptides resulting in a fold increase >1.5 when the treated sample is compared to the control.

| Protein name | Peptide modified sequence | Peptide label | RefQC CV | LOD PAR | CTRL | 1 μM AZD7648 | Fold change |
|---|---|---|---|---|---|---|---|
| sp\|O00762\|UBE2C_HUMAN | GISAFPESDNLFK | UBE2C_pan_GISA | 0.087 | 0.011 | 0.06 | 0.19 | 3.30 |
| sp\|O00762\|UBE2C_HUMAN | LSLEFPSGYPYNAPTVK | UBE2C_pan_LSLE | 0.031 | 0.032 | 0.15 | 0.38 | 2.49 |
| sp\|O60934\|NBN_HUMAN | IPNYQLSPTK | NBN_pan_IPNY | 0.018 | 0.009 | 0.02 | 0.05 | 2.43 |
| sp\|P05412\|JUN_HUMAN | NSDLLTSPDVGLLK | JUN_pan_NSDL | 0.256 | 0.019 | 0.07 | 0.11 | 1.51 |
| sp\|P06400\|RB_HUMAN | IPGGNIYISPLK | RB1_pan_IPGG | 0.025 | 0.005 | 0.04 | 0.07 | 1.64 |
| sp\|P09211\|GSTP1_HUMAN | YISLIYTNYEAGK | GSTP1_pan_YISL | 0.131 | 0.012 | 0.49 | 1.41 | 2.86 |
| sp\|P09874\|PARP1_HUMAN | EELGFRPEYSASQLK | PARP1_pan_EELG | 0.015 | 0.008 | 0.07 | 0.20 | 2.80 |
| sp\|P10909\|CLUS_HUMAN | ASSIIDELFQDR | CLU_pan_ASSI | 0.074 | 0.025 | 0.83 | 2.79 | 3.37 |
| sp\|P12004\|PCNA_HUMAN | DLSHIGDAVVISC[+57]AK | PCNA_pan_DLSH | 0.124 | 0.011 | 9.88 | 15.21 | 1.54 |
| sp\|P12004\|PCNA_HUMAN | DLSHIGDAVVISC[+57]AK[+114]DGVK | PCNA_uK164_DLSH | 0.034 | 0.007 | 0.04 | 0.07 | 1.80 |
| sp\|P12004\|PCNA_HUMAN | LVQGSILK | PCNA_pan_LVQG | 0.066 | 0.016 | 2.80 | 8.17 | 2.92 |
| sp\|P31350\|RIR2_HUMAN | DIQHWESLKPEER | RRM2_pan_DIQH | 0.088 | 0.050 | 0.11 | 0.33 | 3.05 |
| sp\|P31350\|RIR2_HUMAN | IEQEFLTEALPVK | RRM2_pan_IEQE | 0.085 | 0.012 | 0.04 | 0.16 | 4.28 |
| sp\|P38398\|BRCA1_HUMAN | NYPSQEELIK | BRCA1_pan_NYPS | 0.011 | 0.003 | 0.01 | 0.01 | 1.69 |
| sp\|P39748\|FEN1_HUMAN | SIEEIVR | FEN1_pan_SIEE | 0.041 | 0.031 | 0.88 | 1.56 | 1.78 |
| sp\|P54727\|RD23B_HUMAN | IDIDPEETVK | RAD23B_pan_IDID | 0.006 | 0.008 | 3.40 | 9.31 | 2.73 |
| sp\|P54727\|RD23B_HUMAN | ILNDDTALK | RAD23B_pan_ILND | 0.150 | 0.004 | 4.11 | 8.16 | 1.99 |
| sp\|P63279\|UBC9_HUMAN | DHPFGFVAVPTK | UBE2I_pan_DHPF | 0.089 | 0.037 | 4.22 | 13.18 | 3.12 |
| sp\|Q13315\|ATM_HUMAN | NLSDIDQSFNK | ATM_pan_NLSD | 0.061 | 0.012 | 0.23 | 0.42 | 1.82 |

| Protein name | Peptide modified sequence | Peptide label | RefQC CV | LOD PAR | CTRL | 1 μM AZD7648 | Fold change |
|---|---|---|---|---|---|---|---|
| sp\|Q13315\|ATM_HUMAN | SLEISQSYTTTQR | ATM_pan_SLEI | 0.113 | 0.028 | 0.17 | 0.31 | 1.85 |
| sp\|Q8WXE1\|ATRIP_HUMAN | LAAPSVSHVS[+80]PR | ATRIP_pS224_LAAP | 0.024 | 0.003 | 0.01 | 0.01 | 1.75 |
| sp\|Q8WXE1\|ATRIP_HUMAN | LSDGDMTSALR | ATRIP_pan_LSDG | 0.043 | 0.013 | 0.02 | 0.04 | 1.62 |
| sp\|Q92878\|RAD50_HUMAN | LFDVC[+57]GSQDFESDLDR | RAD50_pan_LFDV | 0.237 | 0.008 | 0.01 | 0.05 | 5.50 |
| sp\|Q9BX63\|FANCJ_HUMAN | ATPELGSSENSASSPPR | FANCJ_pan_ATPE | 0.037 | 0.003 | 0.00 | 0.01 | 1.64 |
| sp\|Q9NPD8\|UBE2T_HUMAN | ASQLVGIEK | UBE2T_pan_ASQL | 0.025 | 0.005 | 0.02 | 0.08 | 3.50 |

**Supplementary Table 8. List of all analytes above the assay LOQ identified in PBMCs, collected from patients treated with different doses of AZD7648, following iMRM-MS data analysis.**

The measurements indicated for each analyte represent the ratio between the light versus the heavy peptide, before any normalisation for inputted material and PTPRC (CD45) levels of expression. The table shows, for each analyte, the sequence of the identified peptide, the LOD, the CV of a reference control sample run concurrently with the samples and the amount of material loaded for each sample.

| Protein_Name | Peptide_Modified_Sequence | Analyte_Label | LOQ value | RefQC CV | Patient ID Visit | P1 5mg SCREENING | P1 5mg C1D1 | P1 5mg C1D1 | P2 10mg SCREENING | P2 10mg C1D1 | P2 10mg C1D1 | P2 10mg C1D8/Visit | P3 20 mg SCREENING |
|---|---|---|---|---|---|---|---|---|---|---|---|---|---|
| | | | | | Input Protein Mass by BCA (ug) | 345 | 500 | 500 | 500 | 145 | 500 | 198 | 500 |
| sp\|P68133\|ACTS_HUMAN | AVFPSIVGRPR | ACT_pan_AVFP | 2.569 | ND | | 1.361 | 1.268 | 0.769 | 1.950 | 0.640 | 1.687 | 0.327 | 0.444 |
| sp\|Q13315\|ATM_HUMAN | NLSDIDQSFNK | ATM_pan_NLSD | 0.039 | 0.04 | | 0.188 | 0.201 | 0.136 | 0.244 | 0.107 | 0.072 | 0.073 | 0.123 |
| sp\|Q13315\|ATM_HUMAN | SLEISQSYTTTQR | ATM_pan_SLEI | 0.009 | 0.12 | | 0.347 | 0.273 | 0.221 | 0.199 | | | | |
| sp\|Q8WXE1\|ATRIP_HUMAN | LSDGDMTSALR | ATRIP_pan_LSDG | 0.015 | 0.03 | | 0.440 | 0.370 | 0.270 | 0.196 | 0.055 | 0.052 | 0.028 | 0.124 |
| sp\|P42574\|CASP3_HUMAN | IIHGSES[+80]MDSGISLDNSYK | CASP3_pS26_IIHG | 0.018 | 0.15 | | 0.016 | 0.014 | 0.013 | 0.013 | 0.011 | 0.012 | 0.011 | 0.013 |
| sp\|P06493\|CDK1_HUMAN | VYTHEVVTLWYR | CDK1_pan_VYTH | 0.013 | 0.08 | | 0.335 | 0.259 | 0.167 | 0.183 | 0.034 | 0.027 | 0.016 | 0.077 |
| sp\|P50613\|CDK7_HUMAN | AYT[+80]HQVVTR | CDK7_pT170_AYTH | 0.061 | 0.09 | | 0.389 | 0.329 | 0.205 | 0.206 | 0.107 | 0.498 | 0.024 | 0.148 |
| sp\|O96017\|CHK2_HUMAN | TLC[+57]GTPTYLAPEVLVSVGTAGYNR | CHEK2_pan_TLCG | 0.011 | 0.06 | | 65.161 | 63.426 | 41.770 | 63.528 | 12.199 | 21.304 | 3.137 | 23.213 |
| sp\|P10909\|CLUS_HUMAN | ASSIIDELFQDR | CLU_pan_ASSI | 0.101 | 0.24 | | 0.542 | 0.272 | 0.205 | 0.545 | 0.482 | 0.657 | 0.417 | 0.062 |
| sp\|Q9BX63\|FANCJ_HUMAN | YSTPPYLLEAASHLS[+80]PENFVEDEAK | FANCJ_pS927_YSTP | 0.741 | ND | | 1.207 | 0.744 | 0.627 | 1.375 | 1.346 | 1.786 | 1.123 | 0.211 |
| sp\|P39748\|FEN1_HUMAN | SIEEIVR | FEN1_pan_SIEE | 0.185 | 0.14 | | 4.514 | 3.371 | 2.358 | 4.925 | 0.397 | 0.368 | 0.241 | 2.265 |
| sp\|P04406\|G3P_HUMAN | GALQNIIPASTGAAK | GAPDH_pan_GALQ | 0.126 | 0.19 | | 16.682 | 27.373 | 33.489 | 24.812 | 27.067 | 29.852 | 16.035 | 32.227 |
| sp\|O43561\|LAT_HUMAN | EYVNVS[+80]QELHPGAAK | LAT_pS224_EYVN | 0.006 | ND | | 5.510 | 5.183 | 3.607 | 2.872 | 0.475 | 0.520 | 0.204 | 1.650 |
| sp\|P20700\|LMNB1_HUMAN | AGGPTTPLS[+80]PTR | LMNB1_pS23_AGGP | 0.016 | 0.02 | | | 0.077 | 0.058 | | | | | |
| sp\|P20700\|LMNB1_HUMAN | AGGPTT[+80]PLSPTR | LMNB1_pT20_AGGP | 0.011 | 0.05 | | 0.053 | 0.043 | 0.031 | 0.261 | 0.036 | 0.039 | 0.034 | |
| sp\|Q14566\|MCM6_HUMAN | EIESEIDSEEELINK | MCM6_pan_EIES | 0.084 | 0.10 | | 0.028 | 0.024 | | 0.054 | 0.023 | 0.025 | 0.027 | |
| sp\|Q00987\|MDM2_HUMAN | AISETEENSDELSGER | MDM2_pan_AISE | 0.050 | ND | | | | | | | | | |
| sp\|P49959\|MRE11_HUMAN | IMSQSQVSK | MRE11_pan_IMSQ | 0.333 | 0.02 | | | | | | | | | |
| sp\|O60934\|NBN_HUMAN | IPNYQLSPTK | NBN_pan_IPNY | 0.034 | 0.02 | | 2.881 | 2.697 | 1.917 | 1.765 | | | | 0.864 |
| sp\|P09874\|PARP1_HUMAN | EELGFRPEYSASQLK | PARP1_pan_EELG | 0.059 | 0.03 | | 1.279 | 0.545 | 0.207 | 0.300 | 0.097 | 0.073 | 0.064 | 0.207 |
| sp\|P12004\|PCNA_HUMAN | DLSHIGDAVVISC[+57]AK | PCNA_pan_DLSH | 0.022 | 0.10 | | 14.802 | 13.493 | 9.565 | 14.402 | 2.790 | 5.056 | 0.735 | 5.051 |
| sp\|P12004\|PCNA_HUMAN | DLSHIGDAVVISC[+57]AK[+114]DGVK | PCNA_uK164_DLSH | 0.053 | 0.01 | | 11.508 | 10.666 | 7.327 | 11.495 | 2.173 | 3.924 | 0.574 | 3.785 |
| sp\|P08575\|PTPRC_HUMAN | DPPSEPSPLEAEFQR | PTPRC_pan_DPPS | 0.005 | 0.02 | | 13.306 | 12.232 | 9.900 | 10.609 | 2.007 | 2.143 | 1.100 | 5.152 |
| sp\|P08575\|PTPRC_HUMAN | LFLAEFQSIPR | PTPRC_pan_LFLA | 0.005 | ND | | 11.542 | 43.331 | 39.361 | 86.275 | 10.912 | 14.762 | 23.289 | 14.677 |
| sp\|P54727\|RD23B_HUMAN | IDIDPEETVK | RAD23B_pan_IDID | 0.043 | 0.14 | | | 0.009 | 0.006 | | | 0.003 | 0.001 | |
| sp\|P54727\|RD23B_HUMAN | ILNDDTALK | RAD23B_pan_ILND | 0.017 | 0.08 | | 0.298 | 0.244 | 0.229 | 0.221 | 0.139 | 0.167 | 0.079 | |
| sp\|Q92878\|RAD50_HUMAN | LFDVC[+57]GSQDFESDLDR | RAD50_pan_LFDV | 0.330 | 0.001 | | 0.049 | 0.043 | 0.029 | 0.029 | 0.011 | 0.012 | 0.006 | 0.011 |
| sp\|Q99638\|RAD9A_HUMAN | SPQGPSPVLAEDS[+80]EGEG | RAD9A_pS387_SPQG | 0.064 | 0.07 | | 0.504 | 0.342 | 0.221 | 0.637 | 0.265 | 0.152 | 0.159 | 0.228 |
| sp\|P06400\|RB_HUMAN | IPGGNIYISPLK | **RB1_pan_IPGG** | 0.019 | 0.03 | | 0.439 | 0.307 | 0.179 | 0.690 | 0.300 | 0.180 | 0.184 | 0.301 |
| sp\|Q92541\|RTF1_HUMAN | SASDLSEDLFK | RTF1_pan_SASD | 0.015 | 0.04 | | 0.961 | 0.898 | 0.732 | 0.456 | 0.289 | 0.777 | 0.064 | 0.398 |
| sp\|Q96ER3\|SAAL1_HUMAN | NGAAQPLDQPQEESEEQPVFR | SAAL1_pan_NGAA | 0.113 | 0.11 | | 0.020 | | | 0.017 | | | | |
| sp\|Q15554\|TERF2_HUMAN | DLVLPTQALPASPALK | TERF2_pan_DLVL | 0.009 | 0.07 | | 0.440 | 0.303 | 0.252 | 0.379 | 0.122 | 0.079 | 0.084 | 0.148 |
| sp\|Q15554\|TERF2_HUMAN | DLVLPTQALPAS[+80]PALK | TERF2_pS365_DLVL | 0.019 | 0.05 | | 0.025 | 0.020 | 0.017 | | | | | |
| sp\|Q02223\|TNR17_HUMAN | SLPAALSATEIEK | TNFRSF17_pan_SLPA | 0.003 | 0.081 | | 0.830 | 0.665 | 0.479 | 0.508 | 0.119 | 0.116 | 0.073 | 0.260 |
| sp\|P04637\|P53_HUMAN | ALPNNTSSS[+80]PQPK | TP53_pS315_ALPN | 0.006 | 0.040 | | 1.603 | 1.299 | 0.990 | 1.002 | 0.574 | 0.412 | 0.301 | 0.523 |
| sp\|Q12888\|TP53B_HUMAN | IDEDGENTQIEDTEPMSPVLNSK | TP53BP1_pan_IDED | 0.007 | 0.167 | | 1.515 | 1.112 | 0.793 | 0.834 | 0.162 | 0.183 | | 0.362 |
| sp\|Q12888\|TP53B_HUMAN | IDEDGENTQIEDTEPMS[+80]PVLNSK | TP53BP1_pS552_IDED | 0.084 | 0.111 | | 0.456 | 0.305 | 0.206 | 0.295 | | 0.076 | | 0.133 |
| sp\|P07437\|TBB5_HUMAN | ISVYYNEATGGK | TUBB_pan_ISVY | 3.587 | ND | | | | | | | | | |
| sp\|O00762\|UBE2C_HUMAN | GISAFPESDNLFK | UBE2C_pan_GISA | 0.006 | 0.06 | | 0.040 | 0.036 | 0.026 | 0.025 | 0.012 | 0.016 | | 0.019 |
| sp\|P63279\|UBC9_HUMAN | DHPFGFVAVPTK | UBE2I_pan_DHPF | 0.162 | 0.04 | | 0.199 | 0.132 | 0.094 | 0.098 | 0.068 | 0.049 | 0.057 | 0.056 |
| sp\|Q9NPD8\|UBE2T_HUMAN | ASQLVGIEK | UBE2T_pan_ASQL | 0.007 | 0.04 | | | 1.066 | | | | | | |
| sp\|Q9BVJ6\|UT14A_HUMAN | DSGSQEVLSELR | UTP14A_pan_DSGS | 0.004 | 0.04 | | 0.044 | 0.045 | 0.042 | 0.045 | 0.039 | 0.040 | 0.037 | 0.042 |

| Analyte_Label | P3 20 mg C1D1 | P3 20 mg C1D1 | P4 40 mg SCREENING | P4 40 mg C1D1 | P4 40 mg C1D1 | P4 40 mg C1D8/Visit | P4 40 mg IP DISC | P5 40 mg SCREENING | P5 40 mg C1D1 | P5 40 mg C1D1 | P5 40 mg C1D8/Visit | P5 40 mg C2D1 | P5 40 mg C2D1 | P5 40 mg IP DISC | P6 40 mg SCREENING | P6 40 mg C1D1 | P6 40 mg C1D1 | P6 40 mg C2D1 | P6 40 mg C2D1 | P6 40 mg C1D8/Visit | P7 80mg SCREENING | P7 80mg C1D1 |
|---|---|---|---|---|---|---|---|---|---|---|---|---|---|---|---|---|---|---|---|---|---|---|
| Input Protein Mass by BCA (ug) | 500 | 540 | 471 | 442 | 500 | 230 | 328 | 500 | 550 | 500 | 557 | 500 | 500 | 253 | 500 | 497 | 520 | 366 | 500 | 275 | 295 | 390 |
| ACT_pan_AVFP | 0.755 | 0.757 | 0.903 | 0.719 | 1.038 | 0.754 | 1.392 | 0.573 | 2.533 | 1.717 | 1.141 | 1.538 | 1.876 | 0.311 | 2.620 | 1.523 | 1.613 | 0.590 | 1.596 | 1.426 | 0.580 | 1.150 |
| ATM_pan_NLSD | 0.134 | 0.176 | 0.315 | 0.304 | 0.443 | 0.162 | 0.198 | 0.104 | 0.205 | 0.170 | 0.195 | 0.171 | 0.127 | 0.072 | 0.135 | 0.163 | 0.248 | 0.168 | 0.248 | 0.141 | 0.110 | |
| ATM_pan_SLEI | | 0.172 | | | 0.424 | 0.173 | | | | | | | | | 0.253 | | 0.248 | | 0.300 | | | |
| ATRIP_pan_LSDG | 0.131 | 0.199 | 0.275 | 0.309 | 0.493 | 0.174 | 0.282 | 0.131 | 0.240 | 0.247 | 0.245 | 0.245 | 0.169 | 0.111 | 0.236 | 0.236 | 0.291 | 0.191 | 0.256 | 0.183 | 0.202 | 0.094 |
| CASP3_pS26_IIHG | 0.012 | 0.013 | 0.014 | 0.013 | 0.016 | 0.012 | 0.013 | 0.012 | 0.012 | 0.012 | 0.012 | 0.014 | | 0.011 | 0.013 | 0.013 | 0.017 | 0.012 | 0.017 | 0.013 | 0.013 | |
| CDK1_pan_VYTH | 0.076 | 0.118 | 0.278 | 0.248 | 0.323 | 0.115 | 0.209 | 0.077 | 0.173 | 0.130 | 0.155 | 0.143 | 0.097 | 0.053 | 0.172 | 0.136 | 0.190 | 0.093 | 0.166 | 0.105 | 0.119 | 0.015 |
| CDK7_pT170_AYTH | 0.136 | 0.197 | 0.919 | 1.343 | 1.281 | 0.324 | 0.641 | 0.152 | 0.218 | 0.173 | 0.502 | 0.181 | 0.130 | 0.092 | 0.222 | 0.184 | 0.218 | 0.141 | 0.197 | 0.154 | 0.370 | 1.004 |
| CHEK2_pan_TLCG | 30.230 | 33.900 | 55.282 | 54.870 | 64.142 | 31.520 | 45.738 | 22.179 | 61.122 | 50.506 | 51.644 | 47.751 | 43.661 | 19.408 | 70.344 | 45.818 | 57.284 | 31.002 | 55.829 | 37.354 | 22.814 | 46.468 |
| CLU_pan_ASSI | 0.102 | 0.125 | 0.159 | 0.163 | 0.225 | 0.093 | 0.124 | 0.037 | 0.082 | 0.089 | 0.257 | 0.068 | 0.085 | 0.029 | 0.197 | 0.191 | 0.264 | 0.091 | 0.284 | 0.086 | 0.362 | 0.032 |
| FANCJ_pS927_YSTP | 0.325 | 0.394 | 0.460 | 0.507 | 0.731 | 0.235 | 0.310 | 0.123 | 0.227 | 0.250 | 0.689 | 0.211 | 0.269 | 0.087 | 0.618 | 0.477 | 0.611 | 0.269 | 0.703 | 0.252 | 0.914 | 0.101 |
| FEN1_pan_SIEE | 1.788 | 2.236 | 1.784 | 1.203 | 1.860 | 0.905 | 1.021 | 0.721 | 1.402 | 0.862 | 1.086 | 1.275 | 0.734 | 0.567 | 1.986 | 1.375 | 2.942 | 1.024 | 3.580 | 1.689 | 1.205 | 0.143 |
| GAPDH_pan_GALQ | 31.477 | 27.416 | 9.919 | 12.400 | 12.846 | 9.642 | 12.026 | 9.122 | 12.103 | 10.448 | 7.002 | 6.547 | 9.598 | 1.761 | 15.762 | 30.184 | 19.290 | 24.025 | 21.893 | 13.284 | 6.695 | 12.463 |
| LAT_pS224_EYVN | 1.872 | 2.869 | 3.898 | 3.135 | 4.984 | 2.676 | 4.726 | 1.583 | 3.586 | 3.479 | 3.415 | 3.021 | 2.063 | 1.235 | 3.168 | 3.089 | 4.508 | 2.313 | 3.968 | 2.378 | 4.424 | 1.038 |
| LMNB1_pS23_AGGP | | 0.050 | 0.102 | 0.091 | 0.149 | 0.060 | 0.087 | | 0.064 | | 0.060 | | | | 0.068 | | 0.086 | | 0.066 | | 0.108 | |
| LMNB1_pT20_AGGP | | 0.011 | 0.015 | | 0.010 | 0.016 | 0.013 | | | | | | | | 0.021 | 0.018 | | | 0.022 | 0.018 | 0.063 | |
| MCM6_pan_EIES | | | | | | | | | | | | | | | | 0.010 | | | 0.019 | | 0.020 | |
| MDM2_pan_AISE | | 0.080 | 0.083 | 0.042 | 0.088 | 0.038 | 0.077 | 0.044 | | | 0.131 | | | | 0.079 | | 0.108 | | | 0.061 | | 0.101 |
| MRE11_pan_IMSQ | | | 0.718 | 0.680 | 0.881 | 0.396 | 0.432 | 0.356 | | | | | | | 0.435 | 0.425 | 0.529 | 0.365 | 0.618 | 0.344 | 0.372 | |
| NBN_pan_IPNY | 0.879 | 1.213 | 2.608 | 2.078 | 2.677 | 1.217 | 1.767 | 0.748 | 1.439 | 1.229 | 1.475 | 1.344 | 1.005 | 0.590 | 1.592 | 1.417 | 1.862 | 0.915 | 1.617 | 1.155 | 1.632 | 0.381 |
| PARP1_pan_EELG | 0.198 | 0.211 | 3.791 | 2.036 | 4.429 | 0.652 | 0.688 | 0.107 | 0.295 | 0.320 | 0.378 | 0.283 | 0.185 | 0.146 | 2.599 | 0.633 | 1.132 | 0.338 | 1.080 | 0.339 | 0.444 | |
| PCNA_pan_DLSH | 6.361 | 7.201 | 13.106 | 13.348 | 15.950 | 7.149 | 10.708 | 3.988 | 12.280 | 9.864 | 8.797 | 9.192 | 8.604 | 3.245 | 15.883 | 10.525 | 12.301 | 5.766 | 12.013 | 7.862 | 6.196 | 8.636 |
| PCNA_uK164_DLSH | 5.191 | 5.507 | 9.655 | 9.227 | 11.021 | 5.628 | 8.341 | 3.052 | 9.567 | 7.523 | 6.868 | 7.164 | 6.637 | 2.396 | 12.579 | 8.272 | 9.765 | 4.507 | 9.394 | 6.051 | 4.879 | 6.502 |
| PTPRC_pan_DPPS | 6.418 | 8.181 | 12.397 | 9.425 | 13.976 | 6.343 | 6.303 | 4.435 | 9.448 | 10.220 | 9.487 | 10.801 | 8.222 | 3.687 | 11.285 | 8.226 | 11.650 | 6.428 | 10.913 | 7.170 | 6.001 | 2.141 |
| PTPRC_pan_LFLA | 20.002 | 33.160 | 35.642 | 52.402 | 31.448 | 17.741 | 5.238 | 20.784 | 67.110 | 26.512 | 65.853 | 31.341 | 49.587 | 12.661 | 208.794 | 123.943 | 48.723 | 15.739 | 18.173 | 23.916 | 52.653 | 87.519 |
| RAD23B_pan_IDID | | | 0.003 | 0.004 | 0.004 | | | 0.003 | | 0.006 | 0.007 | 0.005 | 0.004 | 0.004 | | 0.003 | 0.004 | | | | 0.002 | |
| RAD23B_pan_ILND | 0.116 | 0.146 | 0.159 | 0.173 | 0.335 | 0.181 | 0.198 | 0.094 | | 0.139 | 0.182 | 0.218 | 0.143 | 0.084 | 0.195 | 0.181 | 0.248 | 0.132 | 0.320 | 0.153 | | |
| RAD50_pan_LFDV | 0.017 | 0.025 | 0.012 | 0.009 | 0.023 | 0.015 | 0.016 | 0.012 | 0.042 | 0.031 | 0.028 | 0.033 | 0.024 | 0.011 | 0.036 | 0.031 | 0.044 | 0.018 | 0.053 | 0.023 | | |
| RAD9A_pS387_SPQG | 0.233 | 0.309 | 0.378 | 0.389 | 0.670 | 0.249 | 0.301 | 0.146 | 0.280 | 0.279 | 0.266 | 0.349 | 0.182 | 0.094 | 0.425 | 0.309 | 0.534 | 0.202 | 0.511 | 0.195 | 0.301 | |
| RB1_pan_IPGG | 0.273 | 0.334 | 0.301 | 0.427 | 0.746 | 0.221 | 0.264 | 0.136 | 0.275 | 0.215 | 0.259 | 0.332 | 0.175 | 0.081 | 0.467 | 0.381 | 0.582 | 0.259 | 0.592 | 0.219 | 0.287 | 0.011 |
| RTF1_pan_SASD | 0.376 | 0.443 | 1.299 | 1.393 | 1.542 | 0.626 | 1.277 | 0.345 | 0.396 | 0.480 | 0.714 | 0.477 | 0.362 | 0.237 | 0.520 | 0.480 | 0.513 | 0.446 | 0.561 | 0.388 | 1.521 | 0.934 |
| SAAL1_pan_NGAA | | | 0.023 | 0.022 | 0.025 | | | | | | | | | | | | | | | | | |
| TERF2_pan_DLVL | 0.117 | 0.174 | 0.313 | 0.277 | 0.374 | 0.205 | 0.280 | 0.079 | 0.160 | 0.163 | 0.160 | 0.214 | 0.122 | 0.075 | 0.211 | 0.121 | 0.247 | 0.079 | 0.264 | 0.146 | 0.133 | 0.018 |
| TERF2_pS365_DLVL | | 0.016 | | | 0.020 | | | | | | | | | | | | 0.016 | | | | | |
| TNFRSF17_pan_SLPA | 0.280 | 0.384 | 0.646 | 0.665 | 0.955 | 0.376 | 0.487 | 0.206 | 0.444 | 0.493 | 0.504 | 0.480 | 0.311 | 0.178 | 0.512 | 0.416 | 0.633 | 0.322 | 0.588 | 0.330 | 0.498 | 0.145 |
| TP53_pS315_ALPN | 0.593 | 0.839 | 1.509 | 1.793 | 2.097 | 0.683 | 0.941 | 0.545 | 0.953 | 1.023 | 0.951 | 0.949 | 0.648 | 0.348 | 0.921 | | 1.174 | 0.615 | 1.150 | 0.651 | 0.901 | |
| TP53BP1_pan_IDED | 0.372 | 0.617 | 1.644 | 1.770 | 2.150 | 0.684 | 1.110 | 0.353 | 0.822 | 0.757 | 0.789 | 0.736 | 0.516 | 0.280 | 0.827 | 0.777 | 0.979 | 0.504 | 1.010 | 0.578 | 0.678 | 0.143 |
| TP53BP1_pS552_IDED | 0.134 | 0.200 | 0.290 | 0.327 | 0.592 | 0.180 | 0.217 | 0.099 | 0.261 | 0.224 | 0.240 | 0.204 | 0.189 | 0.089 | 0.313 | 0.197 | 0.331 | 0.134 | 0.309 | 0.182 | 0.100 | |
| TUBB_pan_ISVY | | | 5.466 | 4.786 | | | | 4.066 | | 5.158 | 5.214 | 3.751 | 5.905 | 2.594 | 4.461 | 13.495 | 4.971 | | 6.597 | | 3.762 | 2.741 |
| UBE2C_pan_GISA | 0.017 | 0.021 | 0.039 | 0.044 | 0.056 | 0.017 | 0.031 | 0.036 | 0.042 | 0.038 | 0.031 | 0.028 | 0.025 | 0.015 | 0.037 | 0.029 | 0.032 | 0.016 | 0.030 | 0.019 | 0.015 | 0.025 |
| UBE2I_pan_DHPF | 0.041 | 0.060 | 0.109 | 0.143 | 0.214 | 0.086 | 0.116 | 0.038 | 0.091 | 0.077 | 0.092 | 0.106 | 0.055 | 0.031 | 0.143 | 0.100 | 0.140 | 0.059 | 0.136 | 0.074 | 0.079 | 0.016 |
| UBE2T_pan_ASQL | 1.195 | 0.929 | 0.762 | 0.852 | 0.905 | | | 0.766 | 0.689 | 0.673 | 0.692 | 0.695 | 0.770 | 0.665 | | 1.220 | | 1.126 | 1.190 | 1.069 | 0.905 | 0.665 |
| UTP14A_pan_DSGS | 0.042 | 0.043 | 0.055 | 0.055 | 0.058 | 0.046 | 0.045 | 0.047 | 0.048 | 0.047 | 0.049 | 0.045 | 0.044 | 0.042 | 0.042 | 0.043 | 0.048 | 0.042 | 0.044 | 0.042 | 0.039 | 0.042 |

| Analyte_Label | P7 80mg C1D1 | P7 80mg C1D8/Visit | P8 80mg SCREENING | P8 80mg C1D1 | P8 80mg C2D1 |
|---|---|---|---|---|---|
| Input Protein Mass by BCA (ug) | 500 | 579 | 293 | 371 | 327 |
| ACT_pan_AVFP | 2.245 | 3.234 | 0.843 | 1.840 | 1.655 |
| ATM_pan_NLSD | 0.067 | 0.042 | 0.278 | 0.031 | 0.064 |
| ATM_pan_SLEI | | | 0.323 | | |
| ATRIP_pan_LSDG | 0.151 | 0.108 | 0.336 | 0.100 | 0.248 |
| CASP3_pS26_IIHG | 0.011 | 0.012 | 0.015 | 0.012 | 0.011 |
| CDK1_pan_VYTH | 0.089 | 0.068 | 0.200 | 0.068 | 0.158 |
| CDK7_pT170_AYTH | 0.163 | 0.235 | 0.837 | 0.251 | 0.669 |
| CHEK2_pan_TLCG | 44.079 | 42.218 | 53.698 | 42.748 | 50.353 |
| CLU_pan_ASSI | 0.085 | 0.088 | 0.251 | 0.138 | 0.053 |
| FANCJ_pS927_YSTP | 0.240 | 0.244 | 0.686 | 0.442 | 0.172 |
| FEN1_pan_SIEE | 0.809 | 0.338 | 1.066 | 0.421 | 1.270 |
| GAPDH_pan_GALQ | 15.519 | 19.833 | 7.533 | 29.996 | 21.086 |
| LAT_pS224_EYVN | 2.198 | 1.218 | 4.280 | 1.259 | 2.940 |
| LMNB1_pS23_AGGP | | | 0.082 | | |
| LMNB1_pT20_AGGP | 0.040 | 0.015 | 0.021 | 0.051 | 0.060 |
| MCM6_pan_EIES | | | | 0.017 | 0.015 |
| MDM2_pan_AISE | | | 0.040 | 0.085 | |
| MRE11_pan_IMSQ | | | 0.628 | 0.176 | 0.246 |
| NBN_pan_IPNY | 0.848 | 0.713 | 2.037 | 0.546 | 1.625 |
| PARP1_pan_EELG | 0.234 | 0.369 | 0.818 | 0.329 | 0.738 |
| PCNA_pan_DLSH | 9.484 | 10.760 | 11.372 | 9.811 | 12.178 |
| PCNA_uK164_DLSH | 7.182 | 8.018 | 7.760 | 6.528 | 8.120 |
| PTPRC_pan_DPPS | 5.556 | 3.878 | 8.049 | 2.860 | 6.107 |
| PTPRC_pan_LFLA | 60.291 | 34.737 | 36.797 | 141.566 | 68.342 |
| RAD23B_pan_IDID | 0.004 | | 0.003 | | 0.002 |
| RAD23B_pan_ILND | | | 0.256 | | 0.138 |
| RAD50_pan_LFDV | 0.025 | 0.009 | 0.024 | 0.010 | 0.020 |
| RAD9A_pS387_SPQG | 0.147 | 0.079 | 0.312 | 0.222 | 0.274 |
| RB1_pan_IPGG | 0.082 | 0.065 | 0.292 | 0.170 | 0.180 |
| RTF1_pan_SASD | 0.608 | 0.376 | 1.558 | 0.458 | 1.211 |
| SAAL1_pan_NGAA | | | 0.022 | | 0.016 |
| TERF2_pan_DLVL | 0.096 | 0.061 | 0.322 | 0.093 | 0.248 |
| TERF2_pS365_DLVL | | | | | |
| TNFRSF17_pan_SLPA | 0.257 | 0.138 | 0.683 | 0.159 | 0.408 |
| TP53_pS315_ALPN | 0.620 | 0.310 | 1.391 | 0.480 | 0.976 |
| TP53BP1_pan_IDED | 0.353 | 0.367 | 1.308 | 0.564 | 0.999 |
| TP53BP1_pS552_IDED | 0.150 | 0.136 | 0.307 | 0.174 | 0.293 |
| TUBB_pan_ISVY | 14.553 | 3.740 | 5.379 | 7.848 | 2.775 |
| UBE2C_pan_GISA | 0.055 | 0.028 | 0.043 | 0.042 | 0.053 |
| UBE2I_pan_DHPF | 0.047 | 0.033 | 0.159 | 0.026 | 0.096 |
| UBE2T_pan_ASQL | 0.745 | 0.776 | | | 1.075 |
| UTP14A_pan_DSGS | 0.047 | 0.040 | 0.049 | 0.041 | 0.041 |

**Supplementary Figure 1: Geometric mean plasma concentrations (nmol/L) of AZD7648 over time by dosing regimen in A) the Core module and B) the Combination module and C) of PLD in the Combination module (semi-logarithmic scale). LLOQ, lower limit of quantification.**

**A)**

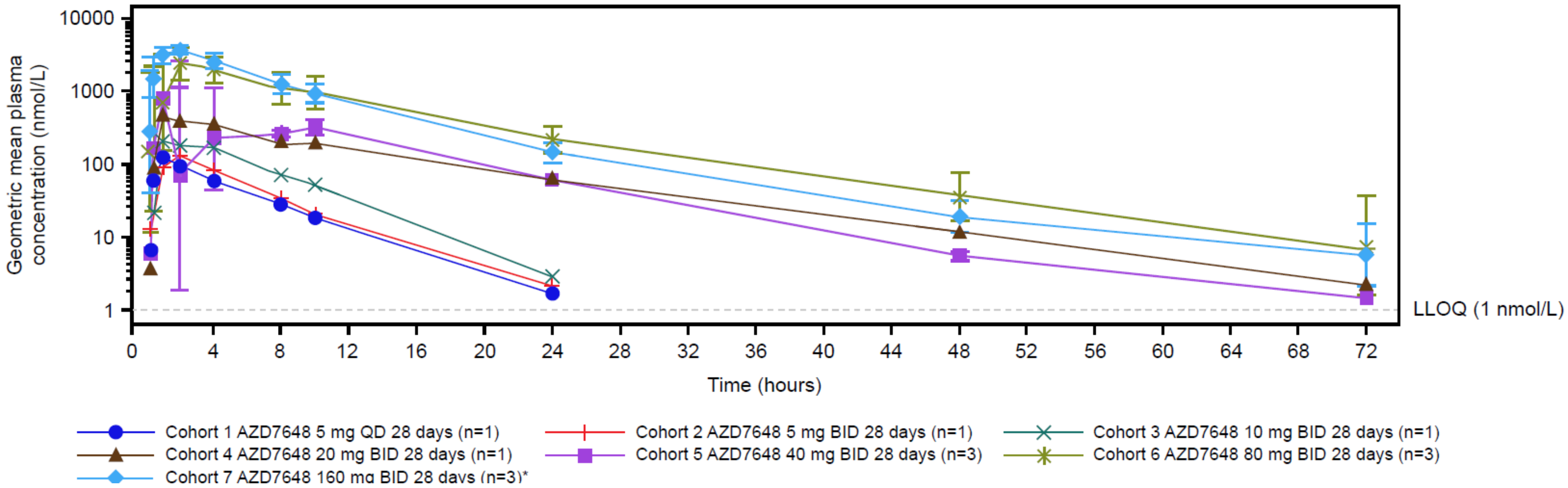

**B)**

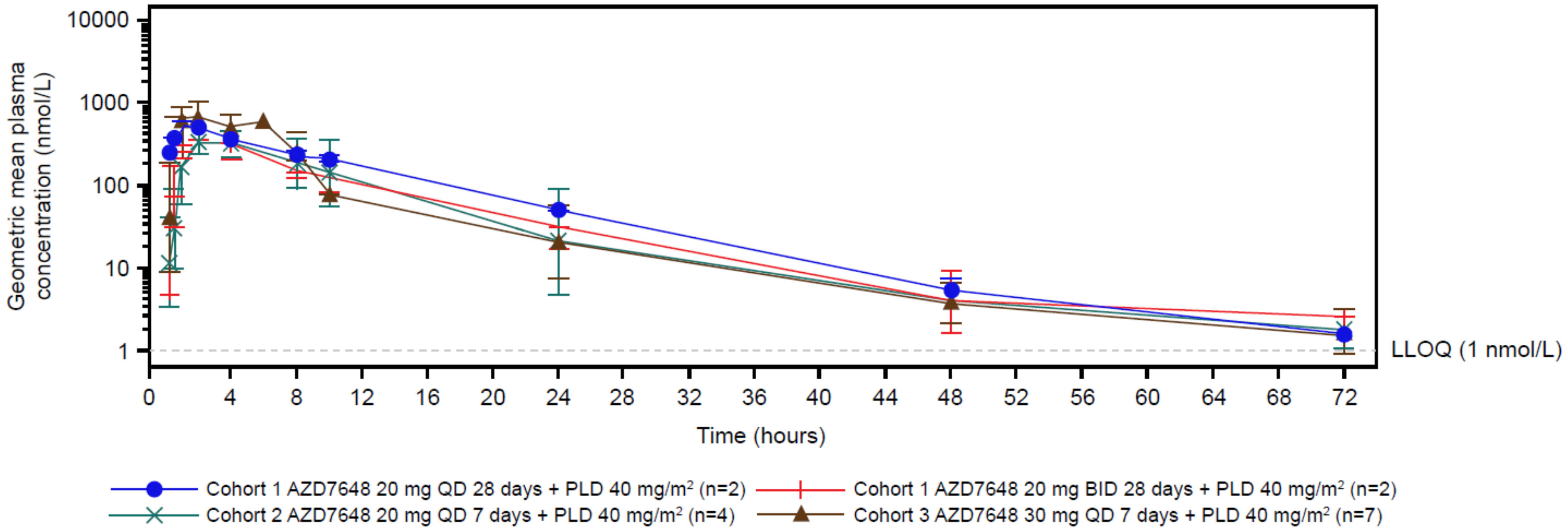

Geometric mean plasma concentration (nmol/L)
10000
1000
100
10
1
LLOQ (1 nmol/L)
0
4
8
12
16
20
24
28
32
36
40
44
48
52
56
60
64
68
72
Time (hours)
Cohort 1 AZD7648 20 mg QD 28 days + PLD 40 mg/m² (n=2)
Cohort 1 AZD7648 20 mg BID 28 days + PLD 40 mg/m² (n=2)
Cohort 2 AZD7648 20 mg QD 7 days + PLD 40 mg/m² (n=4)
Cohort 3 AZD7648 30 mg QD 7 days + PLD 40 mg/m² (n=7)

C)

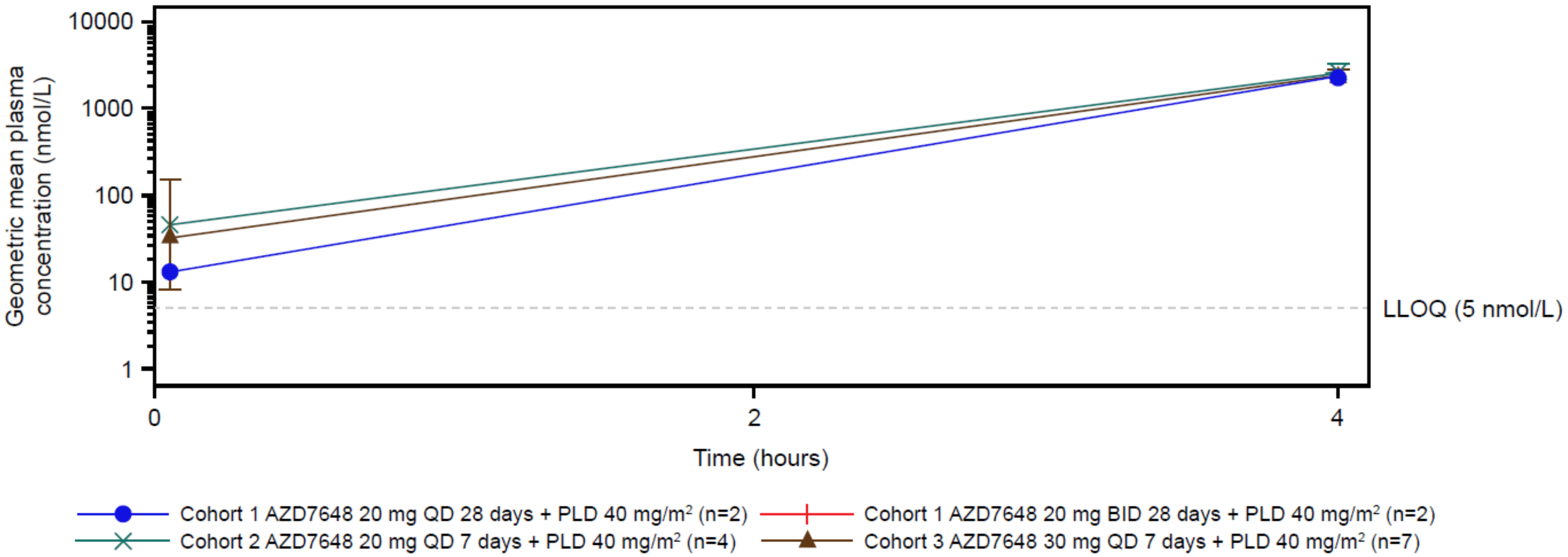

10000
1000
100
10
1
Geometric mean plasma concentration (nmol/L)
LLOQ (5 nmol/L)
0
2
4
Time (hours)
Cohort 1 AZD7648 20 mg QD 28 days + PLD 40 mg/m2 (n=2)
Cohort 1 AZD7648 20 mg BID 28 days + PLD 40 mg/m2 (n=2)
Cohort 2 AZD7648 20 mg QD 7 days + PLD 40 mg/m2 (n=4)
Cohort 3 AZD7648 30 mg QD 7 days + PLD 40 mg/m2 (n=7)

**Supplementary Figure 2: Pharmacodynamic changes in all DDR proteins detected in patient PBMCs at one or more timepoints with AZD7648 monotherapy**

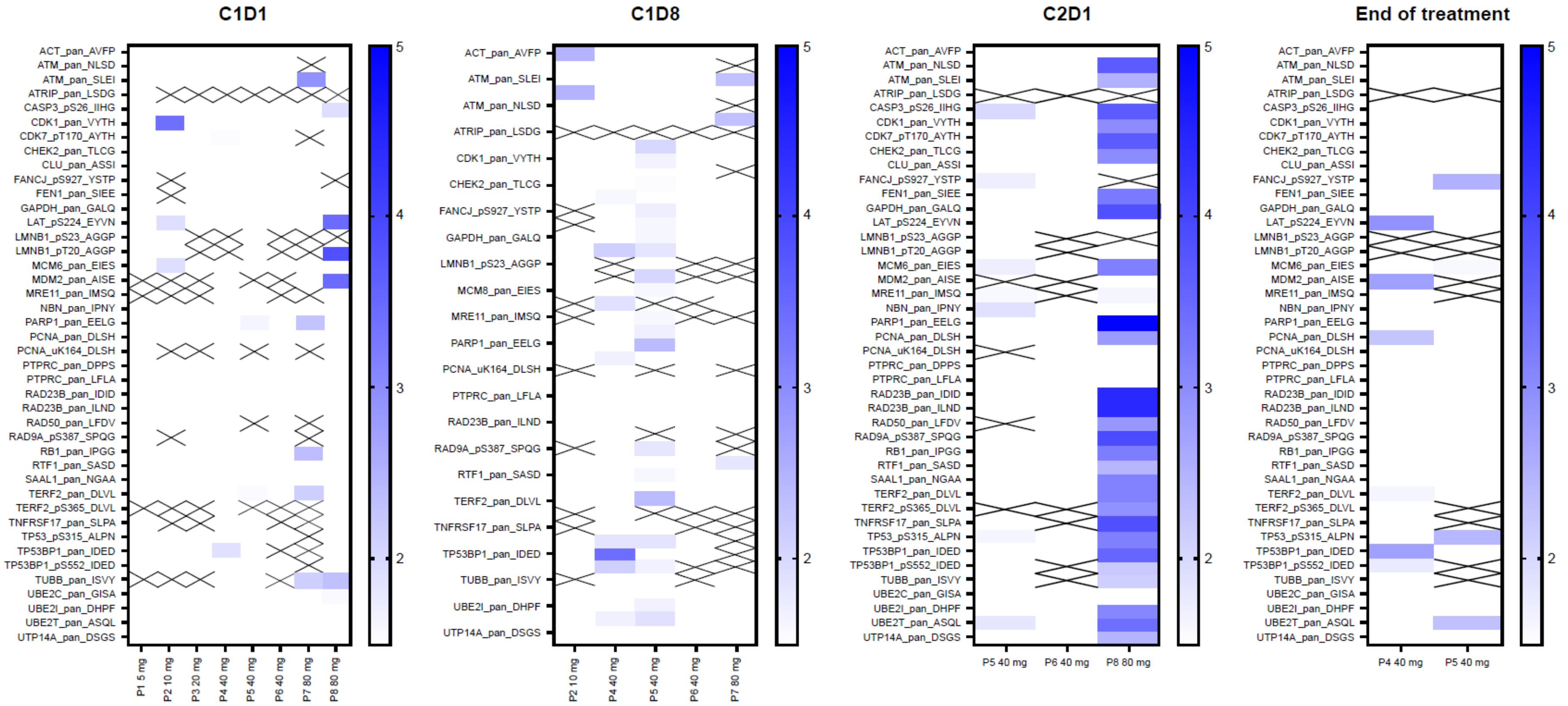

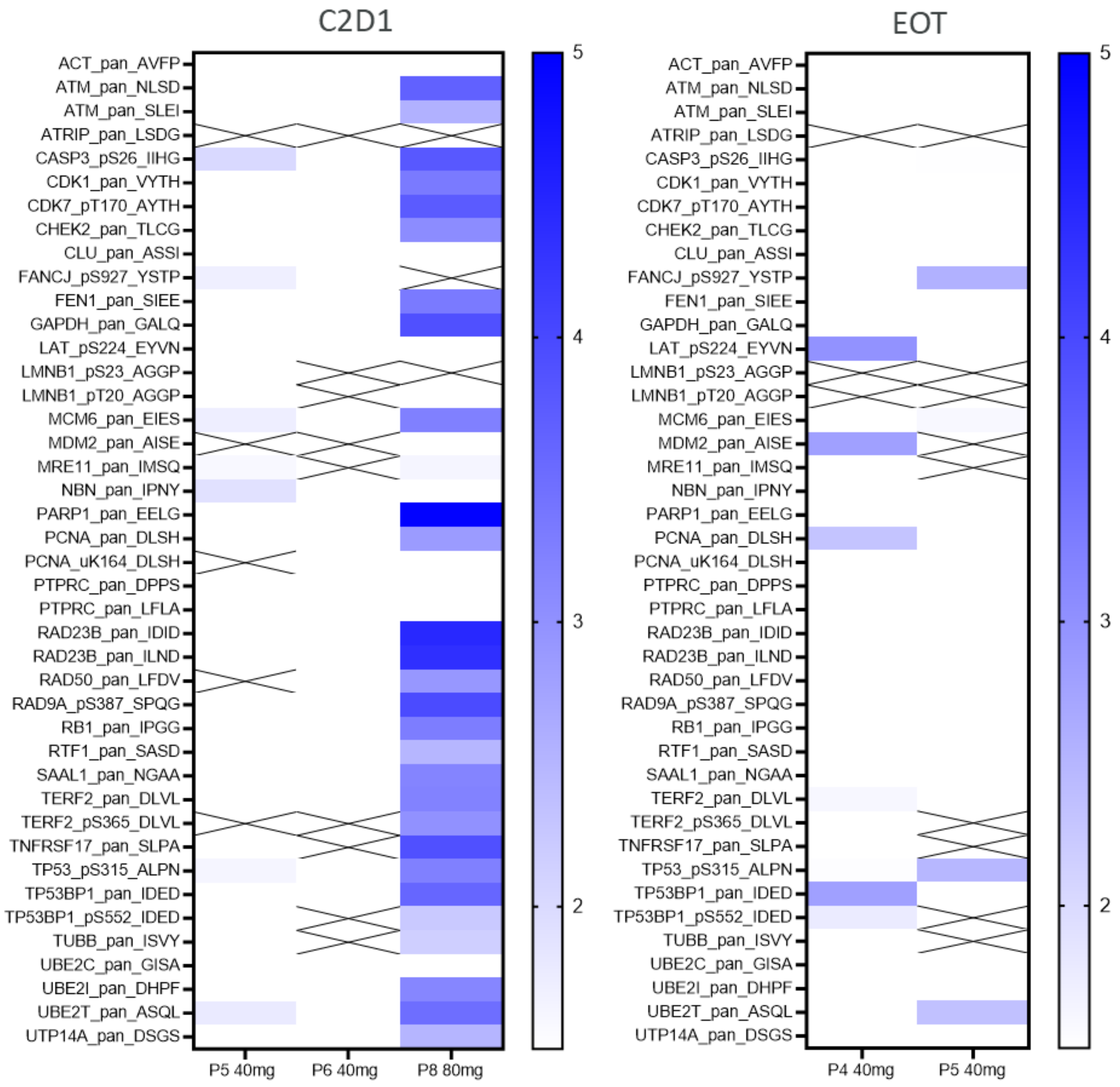
C2D1
EOT
ACT_pan_AVFP
ATM_pan_NLSD
ATM_pan_SLEI
ATRIP_pan_LSDG
CASP3_pS26_IIHG
CDK1_pan_VYTH
CDK7_pT170_AYTH
CHEK2_pan_TLCG
CLU_pan_ASSI
FANCJ_pS927_YSTP
FEN1_pan_SIEE
GAPDH_pan_GALQ
LAT_pS224_EYVN
LMNB1_pS23_AGGP
LMNB1_pT20_AGGP
MCM6_pan_EIES
MDM2_pan_AISE
MRE11_pan_IMSQ
NBN_pan_IPNY
PARP1_pan_EELG
PCNA_pan_DLSH
PCNA_uK164_DLSH
PTPRC_pan_DPPS
PTPRC_pan_LFLA
RAD23B_pan_IDID
RAD23B_pan_ILND
RAD50_pan_LFDV
RAD9A_pS387_SPQG
RB1_pan_IPGG
RTF1_pan_SASD
SAAL1_pan_NGAA
TERF2_pan_DLVL
TERF2_pS365_DLVL
TNFRSF17_pan_SLPA
TP53_pS315_ALPN
TP53BP1_pan_IDED
TP53BP1_pS552_IDED
TUBB_pan_ISVY
UBE2C_pan_GISA
UBE2I_pan_DHPF
UBE2T_pan_ASQL
UTP14A_pan_DSGS
P5 40mg
P6 40mg
P8 80mg
P4 40mg
5
4
3
2
